# Loss of vesicular monoamine transporter 2 in striatum of long COVID and relationship to neuropsychiatric symptoms

**DOI:** 10.1016/j.ebiom.2026.106339

**Published:** 2026-07-10

**Authors:** Yuhan Karida Liu, Devina Persaud, Erica L. Vieira, Joeffre Braga, Pablo Rusjan, Laura Miler, Jennifer S. Rabin, Tina McCluskey, Isabelle Boileau, Thomas Chao, Michael Bagby, Lucas Narciso, Lauren Rose Gray, Neil Vasdev, Kimberly Desmond, Stefan Kloiber, Jerry Warsh, Muhammad Ishrat Husain, Kelly Smart, Wei Wang, Jeffrey H. Meyer

**Affiliations:** a Brain Health Imaging Centre, Campbell Family Mental Health Research Institute, The Centre for Addiction and Mental Health, Toronto, Ontario, Canada; b Department of Pharmacology and Toxicology, University of Toronto, Toronto, Canada; c Department of Psychiatry, McGill University, Montreal, Quebec, Canada; d Department of Psychiatry, University of Toronto, Toronto, Ontario, Canada; e Australian Nuclear Science and Technology Organisation, Sydney, New South Wales, Australia; f Harquail Centre for Neuromodulation, Hurvitz Brain Sciences Program, Sunnybrook Research Institute, Toronto, Ontario, Canada; g Forward Thinking Psychological Services, Vancouver, British Columbia, Canada; h Krembil Brain Institute, University Health Network, Toronto, Ontario, Canada; i Department of Psychological Clinical Science, University of Toronto Scarborough, Toronto, Ontario, Canada; j Division of Neurology, Department of Medicine, Sunnybrook Health Sciences Centre, University of Toronto, Toronto, Canada; k Rehabilitation Sciences Institute, University of Toronto, Canada

**Keywords:** Long COVID, COVID-19, SARS-CoV-2, Dopamine, Vesicular monoamine transporter 2, Positron emission tomography

## Abstract

**Background:**

Dopaminergic neurons are vulnerable to injury from gliosis and have high density of ACE2 receptors, but the integrity of dopaminergic neurons has not been investigated in long COVID. This study examined whether vesicular monoamine transporter 2 (VMAT2) binding, index of dopamine-releasing neuron density, is reduced in the striatum in long COVID and associated with neuropsychiatric symptoms.

**Methods:**

This case–control study (Aug 2022–Apr 2025, Toronto, Canada) included 24 adults with long COVID and 24 age-matched healthy controls, and the healthy control sample was extended to 43 for exploratory analyses. Primary outcome was comparison of (+)[^11^C]DTBZ binding potential (BP_ND_), PET measure of VMAT2 binding, between long COVID and control groups across ventral striatum, dorsal putamen, and dorsal caudate. Secondary outcomes were associations of regional (+)[^11^C]DTBZ BP_ND_ with neuropsychiatric measures (apathy, anhedonia, motor retardation) in long COVID.

**Findings:**

(+)[^11^C]DTBZ BP_ND_ was significantly lower in 24 individuals with long COVID vs 24 age-matched healthy controls (linear mixed effects model, *P* = 4 × 10^−5^; vs 43 controls, *P* = 6 × 10^−4^). Apathy, motor slowing (secondary outcomes) and memory decline (exploratory outcome) correlated with lower (+)[^11^C]DTBZ BP_ND_ in ventral striatum, dorsal putamen and caudate, respectively (|*r*| = 0.48–0.58, *P* = 0.0029–0.018).

**Interpretation:**

Findings of reduced VMAT2 binding may reflect reduced dopaminergic terminal integrity in long COVID. Loss of dopamine nerve terminals may be contributing to symptom correlates of apathy, motor slowing and memory decline suggesting improved function of dopaminergic synapses as a new therapeutic direction to treat long COVID.

**Funding:**

Canadian Institutes of Health Research (191851).

**Research in context**

**Evidence before this study**

We searched in PubMed and EMBASE for publications from database inception to November 2025, using the keywords: ((long COVID) OR (PASC)) AND (brain imaging) OR (postmortem), not limited to English language.

Previous studies in people with long COVID mainly focused on brain inflammation and immune changes. None directly examined whether the brain's dopamine-producing neurons are affected. A previous small post-mortem investigation largely sampling elderly (mean age, 69.7 years; 85% > 60 years) with early changes of dementia who died of SARS-CoV-2 (did not have long COVID) found loss of dopaminergic neurons in the substantia nigra, consistent with previous clinical report that people with Alzheimer's disease and Parkinson's disease are at greater risk of neurological symptoms.

**Added value of this study**

This study suggests a reduction in dopamine-releasing neurons in the brain of people with long COVID. More specifically, (+)[^11^C]DTBZ binding potential (BP_ND_), index of dopaminergic neurons was reduced in striatal regions that participate in motivation, movement speed, and memory. Reduction of (+)[^11^C]DTBZ BP_ND_ correlated with severity of these symptoms.

**Implications of all the available evidence**

These findings suggest that long COVID may involve injury to striatal dopaminergic neurons and that treatments to augment function of dopamine releasing neurons should be tested in long COVID, assessing impact on motivation, motor speed and memory decline.

## Introduction

Long COVID characterised by brain-related symptoms, such as apathy, cognitive slowing, memory difficulties, anhedonia, and low mood, affect about 2% of the general population and persist for months to years.^1^^,^^2^ Despite the high prevalence, no evidence-based treatments have been developed, likely due to an inadequate understanding of core neuropathology. Several neuropathologies have been proposed in long COVID, including loss of synapses, ongoing infection, mitochondrial dysfunction, overexpression of AMPA receptors, sequelae of gut microbiome imbalance and persistent immune response with gliosis.^2^, ^3^, ^4^, ^5^ However, measurements of neuropathological changes correlating with symptoms in the brain of individuals who have long COVID is lacking with most of the focus to date on neuroinflammatory markers.^3^, ^4^, ^5^, ^6^, ^7^, ^8^ Identifying neuropathology in long COVID in humans is difficult because this requires either postmortem brain tissue of long COVID, which is lacking, or brain imaging investigations of specific pathologies.

Although the integrity of dopamine terminals in striatum have not been investigated in long COVID, there are several reasons why they could be vulnerable to injury. First, individuals with long COVID show elevated markers of microglial and astroglial activation in the striatum, with some investigations reporting more widespread elevations.^3^^,^^6^, ^7^, ^8^ Gliosis from excessive synaptic pruning and/or greater generation of reactive oxygen species^9^ may damage dopamine releasing neurons.^10^^,^^11^ Second, direct infection of dopamine releasing neurons innervating the ventral striatum, dorsal putamen and caudate is possible, since their cell bodies in the ventral tegmental area and substantia nigra express a relatively high density of angiotensin converting enzyme 2 receptors.^12^ Third, a preliminary postmortem study of 8 controls and 13 individuals who died of pneumonia from acute COVID-19, albeit some with pathology of early dementia (one with Parkinson's disease) as most were elderly, reported detectable SARS-CoV-2 transcripts and lower dopamine neuronal density in the substantia nigra.^13^ However, it is acknowledged that the latter study^13^ has some limitations with respect to inferring those findings reflect long COVID: Since most participants were elderly, some had pathology of early dementia, including one with Parkinson's disease. Individuals with COVID died months before long COVID could be ascertained. Also, postmortem samples collected during acute COVID-19 illness reflect severe to critical disease, inclusive of coagulopathy and vascular occlusions, in contrast to the vast majority of clinical COVID-19 cases, which are mild to moderate.^1^

The present study applies (+)[^11^C] DTBZ positron emission tomography (PET) in long COVID to study (+)[^11^C] DTBZ Binding Potential (BP_ND_), an index of vesicular monoamine transporter 2 (VMAT2) level and a well-established marker of dopaminergic nerve terminal integrity in the ventral striatum, dorsal putamen, and dorsal caudate.^14^ It was hypothesised that reductions in (+)[^11^C]DTBZ BP_ND_ occur in the ventral striatum, dorsal putamen, and dorsal caudate individuals of long COVID. Furthermore, reductions of (+)[^11^C]DTBZ BP_ND_ in striatal subregions were hypothesised to be associated with specific symptoms: ventral striatum loss with apathy and anhedonia; dorsal putamen loss with motor slowing. Loss of dopamine neurons in these regions in human illness and animal models is associated with these particular symptoms,^15^, ^16^, ^17^ which are also frequent in long COVID.^1^^,^^2^ An exploratory hypothesis was that dorsal caudate loss of (+)[^11^C]DTBZ BP_ND_ is associated with decline in memory. While memory difficulties are more commonly associated with hippocampal and prefrontal cortex injury and may involve pathology across multiple regions, lesions and/or loss of dopamine releasing neurons in the dorsal caudate are also associated with memory problems in humans.^17^

## Methods

### Participants

Twenty-four individuals with long COVID and 24 healthy controls age matched within 5 years were recruited for the main comparison. Additional healthy controls were also recruited, resulting in a total of 43 healthy participants. Participants were recruited from the Greater Toronto Area through clinics at the Centre for Addiction and Mental Health and advertisement. Since the focus of the present study was to investigate (+)[^11^C]DTBZ BP_ND_ in relation to neuropsychiatric symptoms of long COVID, this was defined as emergence of such symptoms from a previously well state within 3 months of acute COVID with symptoms lasting at least 3 months. Acute COVID-19 illness was verified by polymerase chain reaction or rapid antigen testing and was of mild or moderate severity only. All individuals with long COVID experienced apathy and were required to have at least five neuropsychiatric symptoms of a major depressive episode, such as anhedonia, low mood, low concentration, memory decline, and low energy determined by the Structured Clinical Interview for DSM-5 Research Version. Main inclusion criteria for control participants were good health including no history of neurological or psychiatric illness. Exclusion criteria common to all participants were current substance use disorder, lifetime history of moderate or severe substance use disorder, and cigarette or substance use within the previous 2 months. Additional exclusion criterion for individuals with long COVID was a history of neurological illness prior to acute COVID-19.

The matching approach was to first use data from previously scanned healthy as controls, with closest age as the matching criterion. When there were multiple equivalent matches, sex was then chosen as a second criterion. For situations in which there were multiple options after matching for age and sex, the participant with the closest specific activity for their (+)[^11^C]DTBZ PET scan was chosen. Then for the remaining unmatched long COVID participants, healthy controls were recruited who were matched for age.

### Positron emission tomography and magnetic resonance imaging and analyses

370 MBq of (+)[^11^C]DTBZ (±10%) was given intravenously by bolus and scanning was done with a 3-dimensional high-resolution research tomograph PET scanner for 60 min (+)[^11^C]DTBZ is an excellent PET radiotracer for VMAT2 because it has high brain uptake, excellent selectivity, high affinity (Ki = 1 nM), very good specific binding relative to non-displaceable binding (BP_ND_ 1.5–2.5), very good reversibility (time activity curves peak ∼5 min), excellent reliability and is modelled in humans.^18^, ^19^, ^20^ For determining of regions of interest (ROIs), the in-house software ROMI was applied to T1 and PD-weighted brain magnetic resonance images (Discovery MR750 3T scanner, GE Healthcare; 8-channel head coil). In brief, predefined ROIs as previously described^3^^,^^6^ on standard brain template (International Consortium for Brain Mapping/Montreal Neurological Institute 152 MRI) are transformed and deformed (statistical parametric mapping [SPM] normalisation, SPM8; London, UK) to fit each individual MRI followed by iteratively including and deleting voxels based on the probability of each voxel belonging to grey matter (SPM8 segmentation). ROIs were bilateral, weighted by volume. Then, (+)[^11^C]DTBZ BP_ND_ was then determined from the time activity curves extracted from each ROI using the simplified reference tissue model with cerebellar cortex as the reference region (PMOD version 4.2, Bruker Corporation, Fallanden, Switzerland).

### Assessment of long COVID symptoms

Symptoms of apathy, anhedonia, motor speed, and memory, were quantitated with the Marin Apathy Evaluation Scale, Snaith-Hamilton Pleasure Scale, Finger Tapping Test and the Hopkins Verbal Learning Test-Revised respectively. Relationships to additional psychological and neuropsychological tests were explored (Supplementary Table S3).

### Assessment of plasma biomarkers

Analytes related to dopamine metabolism and/or neuronal injury were measured using commercially available ELISA kits (MyBiosource, San Diego, USA) according to the manufacturer's instructions. 3,4-dihydroxyphenylacetic acid (DOPAC), homovanillic acid (HVA), and neurofilament light chain (NFL). All assays were performed in duplicate, and absorbance was recorded using a microplate reader set at 450 nm with wavelength correction at 620 nm. The analytical sensitivity (detection limit) for each assay was: DOPAC (0.1 ng/mL), HVA (1.0 ng/mL), and NfL (2.52 ng/L).

### Ethics

Written informed consent was obtained from all participants. The study was approved by the Research Ethics Board of the Centre for Addiction and Mental Health (REB#201/2022) and Health Canada (CTA#138) prior to the recruiting of the patients. The study was registered on ClinicalTrials.gov with the following reference number: NCT06086366. The study was conducted in accordance with the ethical principles of the 2024 Declaration of Helsinki. We report the study according to the Strengthening the Reporting of Observational studies in Epidemiology (STROBE) reporting guideline.^21^

### Statistics

The primary analysis compared (+)[^11^C]DTBZ BP_ND_ between individuals with long COVID and age matched controls across the primary regions, evaluating a group effect in a linear mixed effects model. Prior to study onset, Power was estimated as higher than 80% for a group comparison of approximately 24 individuals with long COVID vs 24 healthy controls with Cohen's f of 0.37 and a correlation of 0.6 for (+)[^11^C]DTBZ BP_ND_ across regions. This was an interim analysis among stages in the funded grant as future study was intended to extend the sample further to evaluate (+)[^11^C]DTBZ BP_ND_ in subgroups. The actual Cohen's f for the between group difference in the present sampling was 0.65 and the between region correlation is 0.76, so a revised power estimate to detect between group differences for a sample of 24 participants is 99%, or alternatively, for a larger sample of 45 participants as originally planned is approximately 1.00. Also, the effect size of the correlation between ventral striatal (+)[^11^C]DTBZ BP_ND_ and the Marin Apathy Evaluation Scale was *ρ* = −0.54; and the effect size of the correlation between dorsal putamen (+)[^11^C]DTBZ BP_ND_ and the Finger Tapping Test was *r* = 0.51. Hence, based on the data accumulated, the power to detect such correlations for a sample of 24 participants is 0.69, or alternatively for a larger sample of 45 participants as originally planned is >94%.

Correlations between (+)[^11^C]DTBZ BP_ND_ and symptoms applied Pearson Correlation Coefficients, but when assumptions were not met, a Spearman's Rank Correlation Coefficient was applied instead. Comparisons of demographic variables with normal distributions applied independent samples t-tests, but for situations of unequal variances, Welch's test was applied instead. Sex was compared with a chi square test. Ethnicity was compared with Fisher's Exact Test. Exploratory comparisons of symptom severity between groups was done applying a Welch's test due to inequality of variance.

Secondary analyses evaluated relationships between (+)[^11^C]DTBZ BP_ND_ in ventral striatum and the Marin Apathy Evaluation Scale, (+)[^11^C]DTBZ BP_ND_ in ventral striatum and SHAPS and (+)[^11^C]DTBZ BP_ND_ in dorsal putamen with T-scores on the Finger Tapping Test (FFT) with the dominant hand. Exploratory analyses of Pearson correlation coefficients assessed the relationship between (+)[^11^C]DTBZ BP_ND_ in the dorsal caudate and T-scores on the Hopkins Verbal Learning Test-Revised (HVLT-R). Additional symptom measures of interest were the Cognitive Failures Questionnaire (CFQ) as a measure of brain fog and other measures of motor speed like the FFT for the non-dominant hand and speed of stating words on the Stroop Colour Word Test. Symptom measures were controlled for demographic variables if known to be influential, such as age and/or sex. For the primary analysis, a *P* value below 0.05 was considered; for the three secondary analyses, a *P* value below 0.017 was considered significant. The correlation between delayed verbal recall on the HVLT-R and dorsal caudate (+)[^11^C] DTBZ BP_ND_, and the CFQ and ventral striatal (+)[^11^C]DTBZ BP_ND_ are exploratory and uncorrected for number of comparisons. Other results are presented as uncorrected *P* values. These analyses were conducted with SPSS, version 25 (SPSS-IBM).

### Role of funders

The funder had no role in the design and conduct of the study; collection, management, analysis, and interpretation of the data; preparation, review, or approval of the manuscript; and decision to submit the manuscript for publication.

## Results

**Fig. 1:**
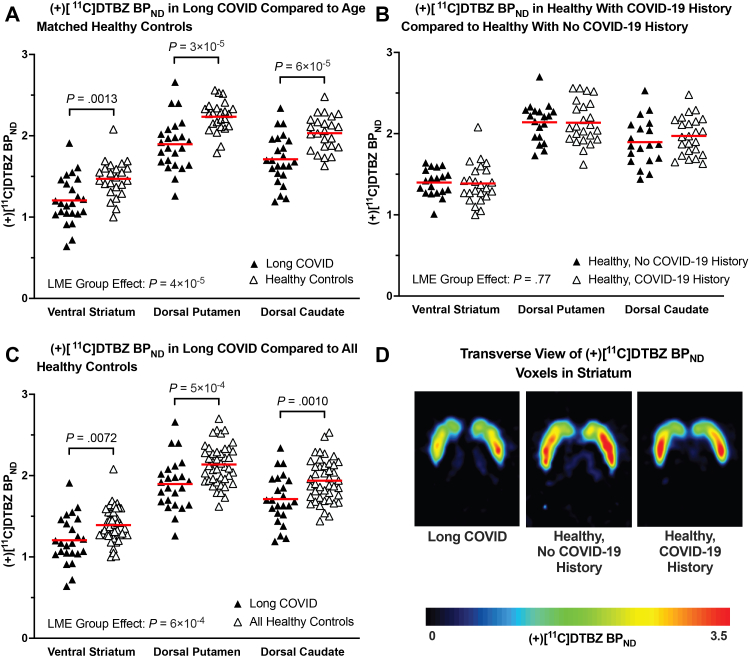
**A to C: Group comparisons applied linear mixed-effects (LME) model with region as an additional factor.** For individual regions, pairwise t-tests were performed on estimated marginal means derived from the model. Red lines represent group means. **D: Representative participant from each group shown.** (+)[^11^C]DTBZ BP_ND_ in voxels quantitated with simplified reference tissue model 2. Colour scale indicates (+)[^11^C]DTBZ BP_ND_ from 0 (black) to 3.5 (red), with reduced (+)[^11^C]DTBZ BP_ND_ observed in the long COVID case compared to both healthy controls.

Twenty-four participants with long COVID (mean [SD] age, 32.2 [10.0] years; 12 [50%] women) and 24 age-matched healthy controls (mean [SD] age, 31.5 [8.9] years; 13 [54%] women) were included in the main analyses (Table 1). The primary outcome was that (+)[^11^C]DTBZ BP_ND_ was lower in all regions assayed in long COVID (LME, effect of group; long COVID mean [SD], 1.60 [0.30]; control mean [SD], 1.91 [0.21]; mean difference, −0.31; 95% CI, −0.44 to −0.17, *P* = 0.000038; Fig. 1A and D, Table 2). The comparison of (+)[^11^C]DTBZ between individuals with long COVID and age-matched healthy controls was also evaluated without the two individuals with a past MDE and results were similar (*P* = 3 × 10^−5^). The healthy control sample was extended (from n = 24 to n = 43; total includes 24 healthy with history of COVID and 19 healthy with no history of COVID) for an exploratory comparison of participants who recovered well from long COVID to those who never had COVID (Fig. 1B and D). These groups had very similar (+)[^11^C]DTBZ BP_ND_ values (LME, effect of group; healthy recovered from COVID mean [SD], 1.83 [0.24]; healthy no history of COVID mean [SD], 1.81 [0.23]; mean difference, 0.02; 95% CI, −0.11 to 0.15, *P* = 0.77). Additional comparison of the 24 participants with long COVID to of the 43 healthy controls yielded similar results (LME, effect of group; long COVID mean [SD], 1.60 [0.30]; control mean [SD], 1.82 [0.24]; mean difference, −0.22; 95% CI, −0.34 to −0.09, *P* = 0.00059; Fig. 1B and D). Injected dose, specific activity and reference tissue radioactivity were similar between individuals with long COVID and healthy controls (Supplementary Table S1, Supplementary Figure S1). There was no evidence for a relationship of (+)[^11^C]DTBZ BP_ND_ to sex, ethnicity, lifetime history of past major depressive disorder (n = 2) or lifetime history of substance use disorder (n = 2, mild past history).

**Table 1:** Demographics of study participants.

Characteristic	Participants, No. (%)		*P* value	All healthy controls (n = 43)	*P* value
	Long COVID (n = 24)	Age-matched healthy controls (n = 24)			
Age, mean (SD), years	32.2 (10.0)	31.3 (9.0)	0.74^a^	29.5 (8.3)	0.24^a^
Sex–Female	12 (50)	14 (58)	0.56^b^	25 (58)	0.52^b^
BMI, mean (SD), kg/m^2^	25.6 (5.6)	23.9 (2.9)	0.20^c^	23.6 (2.7)	0.12^c^
Education, mean (SD), years	16.4 (1.5)	17.2 (3.2)	0.28^c^	17.4 (3.0)	0.07^c^
Ethnicity					
Asian	5 (16.7)	11 (45.8)		20 (44.2)	
Black	2 (8.3)	0		1 (2.3)	
Latin American	2 (8.3)	0		1 (2.3)	
Middle Eastern	1 (4.2)	0		0	
White	13 (54.2)	13 (54.2)		20 (46.5)	
Mixed Heritage	1 (4.2)	0		0	
Ongoing physical symptoms at time of PET scan				1 (2.3)	
Headache	9 (37.5)	NA			
Tiredness	13 (54.2)	NA		NA	
Aches or pains	3 (12.5)	NA		NA	
Muscle aches or pains	5 (20.8)	NA		NA	
Clinical history					
Past MDE prior to COVID-19 infection	2 (8.3)	NA		NA	
History of mild substance use disorder	0	1		2	
Current psychotropic medication	0	0		0	

BMI = body mass index (calculated as weight in kilograms divided by height in metres squared). MDE = major depressive episode. NA = not applicable. PET = positron emission tomography.

a Independent samples t-test.

b Chi-square test.

c Welch's t-test.

**Table 2:** (+)[^11^C]DTBZ BP_ND_ in 24 individuals with long COVID compared to 24 age matched healthy controls.

Region of interest	(+)[^11^C]DTBZ BP_ND_, mean (SD)		% difference^a^	Effect Size	*P* value^b^
	Long COVID (n = 24)	Healthy (n = 24)			
Ventral Striatum	1.21 (0.29)	1.47 (0.23)	20%	1.01	0.0013
Dorsal Putamen	1.90 (0.32)	2.23 (0.19)	16%	1.30	3 × 10^−5^
Dorsal Caudate	1.71 (0.30)	2.03 (0.21)	17%	1.24	6 × 10^−5^

Across regions measured, (+)[^11^C]DTBZ BP_ND_ is significantly lower in 24 individuals with long COVID compared to 24 age matched healthy controls (linear mixed effects model, effect size 1.18, *P* = 4 × 10^−5^).

a % Difference calculated as: [(Healthy mean − Long COVID mean)/((Healthy mean + Long COVID mean)/2)] × 100.

b Comparisons of estimated marginal means.

As SUVR might be applied in clinical settings, this was also calculated over the 30-to-60-time window and similar results were obtained as the SUVR was significantly lower in long COVID (LME, effect of group, *P* = 0.0042). The correlation between SUVR and (+)[^11^C]DTBZ BP_ND_ in the ventral striatum, dorsal caudate and dorsal putamen was 0.89 (95% CI, 0.83–0.93), 0.88 (95% CI, 0.80–0.92) and 0.85 (95% CI, 0.77–0.91), respectively.

**Fig. 2:**
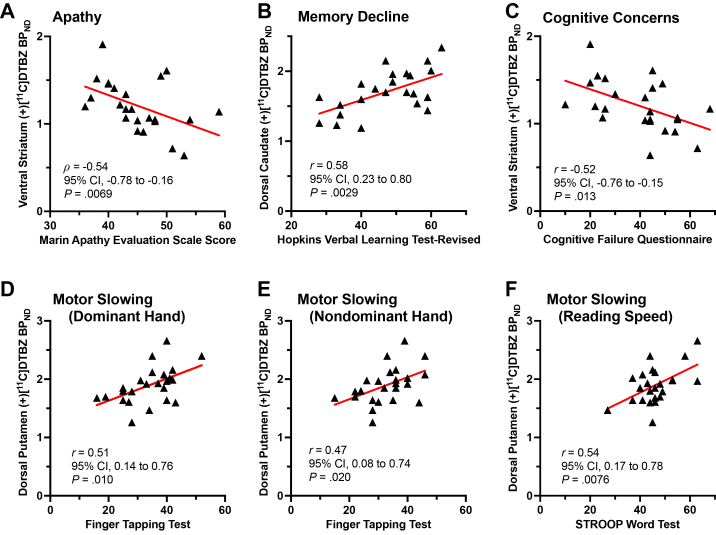
**Correlations between regional (+)[^11^C]DTBZ BP_ND_ and neuropsychiatric test scores (n = 24).** Pearson correlations are presented as *r*, and Spearman correlations are presented as *ρ.* Correlations shown in 2A and 2D were chosen a priori and are below a pre-set threshold but correlations 2B, 2C, 2D, 2E, and 2F were exploratory.

Secondary outcomes were that in participants with long COVID, lower (+)[^11^C]DTBZ BP_ND_ in the ventral striatum correlated with greater symptom severity on the Marin Apathy Evaluation Scale (AES) (*r* = −0.54; 95% CI, −0.78 to −0.16; *P* = 0.0069; Fig. 2A) and lower (+)[^11^C]DTBZ BP_ND_ in the dorsal putamen correlated with slower motor speed measured with the Finger Tapping Test (FTT) (*r* = 0.51; 95% CI, 0.14–0.76; *P* = 0.010; Fig. 2D). (+)[^11^C]DTBZ BP_ND_ in ventral striatum did not correlate with greater symptom severity on the Snaith-Hamilton Pleasure Scale (SHAPS) (*r* = 0.079; 95% CI, −0.47 to 0.34; *P* = 0.71).

Exploratory outcomes included that lower (+)[^11^C]DTBZ BP_ND_ in the dorsal caudate correlated with worse memory performance, as measured with the Hopkins Verbal Learning Test-Revised (HVLT-R) Delayed Recall (*r* = 0.58; 95% CI, 0.23–0.80; *P* = 0.0029; Fig. 2B) and lower (+)[^11^C]DTBZ BP_ND_ in the ventral striatum correlated with the Cognitive Failures Questionnaire (CFQ) (*r* = −0.52; 95% CI, −0.76 to −0.15; *P* = 0.013; Fig. 2C). Performance on other tests of motor speed like the Finger Tapping Test of the non-dominant hand, and speed of stating words on the Stroop Colour Word Test correlated with (+)[^11^C]DTBZ BP_ND_ in the dorsal putamen (respectively: *r* = 0.47; 95% CI, 0.08–0.74; *P* = 0.020; Fig. 2E; *r* = 0.54; 95% CI, 0.17–0.78; *P* = 0.0076; Fig. 2F). Correlations were negligible between (+)[^11^C]DTBZ BP_ND_ in all regions and the Beck Depression Inventory (Supplementary Figure S2), predominant SARS-CoV-2 variant in Ontario, Canada and the interval between scanning and time of acute COVID-19 illness predating long COVID symptoms (range 2–56 months, first quartile 13, median 22, and third quartile 30.5, Supplementary Figure S3). Correlations of (+)[^11^C]DTBZ BP_ND_ with blood serum markers of dopamine or neuronal injury in the long COVID group were also negligible (Supplementary Figure S4). Exploratory analyses evaluating relationship of regional (+)[^11^C]DTBZ BP_ND_ and neuropsychological testing were also conducted (Supplementary Table S3).

The symptoms assessed for correlation with lower regional (+)[^11^C]DTBZ BP_ND_ also differed between individuals with long COVID (n = 24) and healthy controls (n = 24) who recovered well after COVID-19. Welch's t-test, Marin Apathy Evaluation Scale (mean difference, 21.7; 95% CI, 18.8–24.6, *P* < 0.000001), Finger Tapping Test Dominant Hand (mean difference, −10.9; 95% CI, −15.9 to −5.5, *P* = 0.0001), Finger Tapping Test Non-Dominant Hand (mean difference, −11.2; 95% CI, −16.3 to −6.1, *P* = 0.0001), Hopkins Verbal Learning Test–Revised Delayed Recall (mean difference, −10.9; 95% CI, −15.6 to −6.2, *P* = 0.00006), and Cognitive Failures Questionnaire (mean difference, 17.3; 95% CI, 9.6–25.0, *P* = 0.00005) (Supplementary Table S2).

## Discussion

The first main finding of this investigation was lower striatal (+)[^11^C]DTBZ BP_ND_ in long COVID. The other main findings were correlations between lower (+)[^11^C]DTBZ BP_ND_ in ventral striatum, dorsal putamen and caudate respectively with symptoms of apathy, motor slowing and memory decline. These findings may have important implications for the pathophysiology of several impactful symptoms of long COVID and suggest new avenues for treatment. Our enrolment criterion of not having a pre-existing neurological disorder, age 18 to 75, non-smoking and being in a well state just prior to the episode of COVID likely resulted in a younger mean age of the long COVID sample than the mean age of long COVID in the general population But this is a trade-off for an important advantage because these biases were avoided enabling clearer interpretation of the effect of being in either the long COVID or healthy group on (+)[^11^C]DTZB BP_ND_.

Approximately 95% of VMAT2 binding in striatum is contained within dopamine releasing neurons^22^ so a reduction in (+)[^11^C]DTBZ BP_ND_ is inferred to represent a loss of dopamine releasing neurons. This interpretation is consistently applied as VMAT2 PET imaging is a well-established method to detect pattern and progression of loss of dopamine nerve terminals in neurodegenerative illnesses and prodromal states like Parkinson's Disease, progressive supranuclear palsy, and rapid eye movement disorder.^23^, ^24^, ^25^ While these findings reflect support for loss of dopaminergic neurons in long COVID, an important consideration is whether it is plausible that the specific regional reductions in loss of dopamine releasing neurons are aetiologically implicated in the correlated symptoms. Among the symptoms correlated with regional (+)[^11^C]DTBZ BP_ND_, apathy is the most frequent in long COVID, but memory difficulties, motor slowing, and cognitive concerns are also highly prevalent.^1^ Electrophysiology, optogenetic, and viral tracing studies demonstrate that subsets of dopamine releasing neurons in nucleus accumbens participate in motivation and cognition; subsets in dorsal putamen participate in motor control and acceleration; and subsets in caudate participate in memory retention.^15^^,^^16^ Injury to dopamine releasing neurons in these regions through neurological illness and toxin exposure in humans or corresponding models in animals are also associated with impairments of these functions.^17^ The magnitude of reduction in regional (+)[^11^C]DTBZ BP_ND_ associated with clinical level symptoms in the present study may be sufficient for apathy and decline in delayed verbal memory, given that such magnitudes are comparable to mild to moderate Parkinson's disease,^23^ in which these symptoms occur.^26^ Similarly, the magnitude of reduction in dorsal putamen (+)[^11^C]DTBZ BP_ND_ associated with impairment on the Finger Tapping Test is comparable to that observed in REM sleep behaviour disorder,^24^ a frequent prodrome of Parkinson's disease associated with reduced performance on this test.^27^

Given that 25% of the variance in symptoms of apathy, motor slowing, and memory decline was associated with loss of (+)[^11^C]DTBZ BP_ND_, the present study makes a case for a hypothesis that interventions targeting dopamine releasing neurons in long COVID would have therapeutic impact. This is a notable shift from present therapeutic development, given that no current trials focus on augmenting functions of dopamine releasing neurons. Several therapeutics could be repurposed in human clinical trials to assess this hypothesis, including dopamine precursors such as l-dopa or inhibitors of dopamine metabolism like monoamine oxidase inhibitors. These interventions would enhance tonic dopamine release broadly, and phasic release in intact synapses, thereby reducing impact of lost synapses. It is important to consider that dopaminergic augmentation can have significant side-effects and might only be appropriate for individuals with long COVID with a dopaminergic phenotype. We presented a number of exploratory analyses, and whether such results may be statistically meaningful merits discussion. As (+)[^11^C]DTBZ BP_ND_ is highly correlated across different brain regions, there are approximately 12 independent correlations assessed in relation to (+)[^11^C]DTBZ BP_ND_. Hence, uncorrected *P*-values of less than 0.004 may be of greater interest for future hypotheses whereas uncorrected *P* -values of greater than 0.004 are less likely to be of interest for future hypotheses.

In addition, while low-cost peripheral plasma biomarkers may offer complementary insight, their relationship to striatal dopamine measures should be interpreted cautiously. Major sources of blood dopamine include release from chromaffin cells in the adrenal medulla, the sympathetic nervous system, and decarboxylation-active cells in the kidney and the mesentery, so contributions from the striatum represent only a small fraction.^28^ Likewise, the absence of a relationship between peripheral NfL and striatal (+)[^11^C]DTBZ BP_ND_ can be reasonably explained by the fact that NfL reflects neuronal injury to both central and peripheral neurons, so the striatum may not adequately reflect the overall source. Timing of injury relative to the assay may also be important, so this may be a measure best obtained, even if logistically difficult, during acute COVID-19.^29^

(+)[^11^C]DTBZ PET was chosen because the imaging agent has excellent qualities (such as high selectivity, high affinity, and high reversibility) and has been modelled in humans. BP_ND_ was chosen because it is highly quantitative. While SUVR is less quantitative, this measure yielded similar results to (+)[^11^C]DTBZ BP_ND_. The results applying SUVR suggest a promising direction for future clinical translation, moving from ^11^C PET to the longer radioactive half-life of ^18^F PET. For example, the use of SUVR in conjunction with a fluorinated PET imaging agent like [^18^F]AV133, would enable multiple individuals to be scanned after a single synthesis of imaging agent, reducing costs.^30^ In addition, production of a fluorinated imaging agent would enable centralised production of the imaging agent at a site with a cyclotron which could then be distributed to multiple centres, including those without a cyclotron. This would make PET measurement of VMAT2 binding more feasible in clinical settings to either stratify potential participants for clinical trials or, once treatments are developed, to use a personalised medicine approach to match individuals to optimal treatment.

Also, long COVID is heterogeneous and the individuals of long COVID in the present study have symptoms of apathy, cognitive concerns, difficulty with delayed memory recall and motor slowing (Supplementary Table S2). These symptoms of long COVID are likely to be relevant to findings given that they were not present prior to having COVID, and that they correlated with regional (+)[^11^C]DTBZ BP_ND_. These symptoms, in particular apathy and cognitive concerns, are common in long COVID, but some individuals with long COVID do not have these symptoms and accordingly, it is unknown in the absence of these symptoms whether such individuals with long COVID would also have low (+)[^11^C]DTBZ BP_ND_. In addition, since study participants experienced depressive symptoms the question of whether the findings are consequent to depressive symptoms could be considered, however, there was no correlation between the Beck Depression Inventory and (+)[^11^C]DTBZ BP_ND_ so this is unlikely. Moreover, only two participants had a history of major depressive episodes prior to COVID-19 and main results comparing individuals with long COVID to controls was similar regardless of removal of these two participants from the analyses.

A limitation is that, although it is plausible that the relationships of loss of (+)[^11^C]DTBZ contribute to symptoms, human imaging studies identify correlations and do not prove causality. Other effects of SARS-CoV-2 may also contribute to the correlated symptoms, particularly for symptom measures that reflect a broader measurement of brain function like the Cognitive Failures Questionnaire. An approach to determine the relative impact of loss of dopamine neurons vs other mechanisms would be the degree of symptom reduction found through restoration of synapses of dopamine releasing neurons to premorbid function is accompanied by concurrent symptom recovery. This is challenging because full restoration of presynaptic dopamine releasing neurons is not well established in clinical settings but the impact of treatments that augment dopamine release may provide further insight. A second limitation is that even though (+)[^11^C]DTBZ BP_ND_ is an established proxy for presynaptic dopaminergic terminal integrity, it is an index of VMAT2 density. As such it could reflect reduced concentration of dopamine vesicles per presynaptic neuron, a reduction in VMAT2 density per dopamine containing vesicle or other related regulatory processes rather than a structural reduction of dopamine releasing synapses, although this is not typically observed in neuropsychiatric illness. Even so, the translational implications may be similar, with focus on optimising the kinetics of extracellular dopamine released at synapses. Other limitations are that findings represent a specific, albeit important phenotype of long COVID, a larger sample size could have been assayed; that control groups were healthy, and a psychiatric control group was not included; that other mechanisms might theoretically lead to the difference in (+)[^11^C]DTBZ BP_ND_ between health and long COVID. Examples of the latter would be that a symptom of long COVID leads to change in (+)[^11^C]DTBZ BP_ND_ or that lower (+)[^11^C]DTBZ BP_ND_ was present prior to onset of long COVID. Finally, sensitivity of in vivo PET measurement to detect changes in illness requires a substantial level of specific binding relative to non-displaceable binding, so only brain regions amply meeting this criterion were assayed.

In conclusion, (+)[^11^C]DTBZ BP_ND_ were lower in the ventral striatum, dorsal putamen and dorsal caudate in adults with long COVID. Lower ventral striatal (+)[^11^C]DTBZ BP_ND_ correlated with apathy and cognitive concerns. Lower dorsal putamen (+)[^11^C]DTBZ BP_ND_ correlated with motor slowing and lower dorsal caudate (+)[^11^C]DTBZ BP_ND_ correlated with worse verbal memory performance. These symptoms that correlated with reductions in (+)[^11^C]DTBZ BP_ND_ are also important neuropsychiatric symptoms of long COVID. The magnitude of (+)[^11^C]DTBZ BP_ND_ loss in long COVID is similar to other illnesses with common pathology and symptoms, such as the ventral striatum and caudate of mild to moderate Parkinson's disease and the dorsal putamen of REM sleep behaviour disorder).^23^^,^^24^^,^^26^ Given the heterogeneity of long COVID, future studies in larger, similarly well-controlled cohorts would help confirm the robustness and generalisability of these findings. Such studies could also test whether interventions aimed at restoring dopamine-releasing synapses and/or augmenting tonic and phasic dopamine release in the ventral striatum, dorsal putamen, and dorsal caudate influence correlated symptoms in long COVID.

## Contributors

YKL and JHM had full access to all the data in the study and take responsibility for the integrity of the data and the accuracy of the data analysis. YKL and JHM contributed to the conception and design of the study. All authors contributed to data acquisition, analysis, or interpretation. YKL, DP, and JHM drafted the manuscript, and all authors critically revised it for important intellectual content. YKL, LM, and JHM performed the statistical analysis. All authors provided administrative, technical, or material support. JHM, IB, NV, MB, WW, KD, KS, MIH obtained funding. All authors have reviewed the final version of the manuscript and agreed to its submission for publication.

## Data sharing statement

Deidentified data corresponding to Figs. 1 and 2 will be made available in a Supplementary Appendix at the time of publication.

## Declaration of interests

JHM has patents for a dietary supplement to prevent postpartum blues and postpartum depression and is received funding from CAMH and Exeltis for that research. He also owns stock in Moderna. In mid January 2026, JHM has applied for patenting for rasagiline and a dopamine precursor approach of tyramine use for an indication of long COVID. The patent is pending approval as of May 19, 2026. All other authors report no biomedical financial interests or potential conflicts of interest. No other disclosures were reported.

## Appendix A Supplementary data

Supplementary Tables

Protocol
