# Supplementary material for: Loss of vesicular monoamine transporter 2 in striatum of long COVID and relationship to neuropsychiatric symptoms
Source: eBioMedicine. 2026 Jul 10;130:106339. doi: 10.1016/j.ebiom.2026.106339 (PMC13476897; doi:10.1016/j.ebiom.2026.106339)
Supplement: Supplementary file 1 [file mmc3.pdf]

Presynaptic Imaging in Major Depressive Episodes After COVID-19

Protocol Version 4.0

2023 02 10

CAMHPET-CTA-138

REB #: 2022/201

Key Roles and Contact Information

**Principal and Qualified Investigator:**	Dr. Jeffrey H. Meyer, *MD PhD FRCPC*<br><br>Centre for Addiction and Mental Health<br>250 College Street<br>Toronto, ON, M5T 1R8<br>(416) 535-8501 x34007
**Co-Principal Investigator:**	Not applicable
**Co-Investigators:**	Dr. Michael Bagby, *PhD*<br>Dr. Stephen Kish, *PhD*<br>Dr. Neil Vasdev, *PhD*<br>Dr. Kimberly Desmond, *PhD*<br>Dr. Kelly Smart, *PhD*<br>Dr. Wei Wang, *PhD*
**Medical Expert**<br>**And Co-Investigator:**	Dr. Ishrat Husain, *MBBS MD MRCPsych*<br><br>Centre for Addiction and Mental Health<br>100 Stokes Street<br>Toronto, ON, M6J 1H4<br>(416) 535-8501 x37838
**Sponsor:**	Brain Health Imaging Centre<br><br>Centre for Addiction and Mental Health<br>250 College Street<br>Toronto, ON, M5T 1R8<br>(416) 535-8501 x36798
**Monitor:**	Quality Assurance, Centre for Addiction and Mental Health<br><br>Centre for Addiction and Mental Health<br>250 College Street<br>Toronto, ON, M5T 1R8<br>(416) 805-0647
**Funding Agency:**	Canadian Institutes of Health Research

Qualified Investigator Statement of Compliance

This document is a protocol for research involving human participants and will be conducted in line with the International Conference on Harmonisation Good Clinical Practice E6 (ICH-GCP), Declaration of Helsinki and applicable Regulatory Ethics Board at the Centre for Addiction and Mental Health (CAMH REB). The signature below denotes confirmation that this research study will be conducted according to all stipulations of the protocol, and according to local legal and regulatory requirements and ICH-GCP guidelines.

Jeffrey Meyer Digitally signed by Jeffrey Meyer Date: 2023.02.14 11:28:11 -05'00'

February 14, 2023

Signature — Date (yyyy-mmm-dd)

Dr. Jeffrey H. Meyer
PI/QI
Brain Health Imaging Centre
Centre for Addiction and Mental Health

INTRODUCTION AND BACKGROUND

1.1 Importance of Problem to be Addressed

1.1.1 Definitions: COVID-19 is defined as the clinical syndrome consequent to infection by severe acute respiratory syndrome coronavirus 2 (SARS-CoV-2)[1]. Post-acute sequelae of SARS-CoV-2 infection (PASC) is a broad term that includes all forms of persistent symptoms after an acute infection whereas this proposal focuses on a specific subset of persistent symptoms. In this proposal, COVID-DNP is a short form for a major depressive episode (MDE) with or without additional neuropsychiatric symptoms with onset within 3 months after COVID-19 with mild to moderate respiratory symptoms. (*Note:* During COVID-19, mild to moderate respiratory symptoms occur in ~90% to 95% of omicron BA.1 cases and newer variants[2-5] and 80% to 85% of cases from the initial SARS CoV-2 strain[6, 7]).

1.1.2 COVID-19 is Infecting a High Proportion of the United States and the World: At least 25% of Americans have tested positive for SARS-CoV-2[8]. COVID-19 is also a major international problem such that prevalence rates of previous positive tests in countries with monitoring capacity typically exceed ~25%[9]. These are generally believed to be underestimates of lifetime prevalence.

1.1.3 After the Acute Respiratory Illness of COVID-19 Ends, There is A High Prevalence of Subsequent COVID-DNP, Even After Infection with Omicron Variants in Vaccinated People: After respiratory symptoms of COVID-19 subside, for most SARS-CoV2 variants, the prevalence rate of COVID-DNP is ~15 to 30% [10-12] and ~10% report cognitive impairment [10-12]. The risk of COVID-DNP in those triple vaccinated is about 8% regardless of whether the strain is Delta or Omicron BA.1 and the risk of such symptoms is about 20% higher from Omicron BA.2 than Omicron BA.1, according to a British study of over a million participants[13]. In general, being fully vaccinated may reduce the risk of having persistent symptoms by about 25% [14-16], and newer variants have a lower risk of persistent symptoms by about 25% [17, 18], (totalling to ~50% lower risk compared to those unvaccinated with early strains) but newer variants are more transmissible, and reinfection is associated with a greater risk of subsequent depressive symptoms[19]. Long term persistence of MDE is heterogeneous[20]. Some symptoms may last months[21], but, symptoms like anhedonia, amotivation, low energy, motor retardation and cognitive impairment often persist much longer[20, 22-26]. So COVID-DNP, the focus of this proposal, continues to be a major public health problem[27, 28].

1.2 Neuropathology of Gliosis Is Implicated in Postmortem Study of COVID-19

1.2.1 There are plausible mechanisms through which COVID-19 Could Cause Neuroinflammation and Gliosis: One mechanism is viral uptake: Viral uptake into the central nervous system could occur via the olfactory[1, 29], trigeminal and vagal nerves[30]; or via circumventricular organs that are missing some of the protective junctional proteins of the blood brain barrier[30]; or via infection of endothelial cells in brain vasculature[29] which may reduce blood brain barrier protectiveness[31], such as altering expression of tight junction proteins. ACE2 receptors, an entry point of viral uptake, are expressed in neurons and glia[31, 32]. A second

mechanism through which SARS-CoV-2 may cause central inflammation and gliosis is through causing peripheral inflammation[29]. The peripheral inflammatory system communicates with the central nervous system through mechanisms like cytokine stimulation of peripheral nerves; macrophage like cells proximal to the ventricles secreting cytokines; and transport of cytokines across the blood brain barrier[33]. Highly elevated cytokines may occur during acute COVID-19, which, at its extreme termed a "cytokine storm"[29]. A third mechanism, after acute severe COVID-19 (unlikely relevance for this proposal of COVID-DNP after mild to moderate COVID-19) is induction of neuroinflammation after coagulopathy and/or hypoxia[29, 34].

1.2.2 Postmortem samples show neuroinflammatory changes of microglial and/or astroglial activation in brain regions assayed in those who died of SARS-CoV-2[30, 35]. For example, the largest dataset in a single study of 43 postmortem cases found microglial and astroglial activation to be present in almost all cases in most brain regions sampled[36] and a review of 45 postmortem studies report that about half of the 285 cases find evidence of microglial and astroglial activation[37]. This is consistent with laboratory conditions of SARS-CoV-2 exposure to non-human primates in which a sample of 6 exposed compared to 6 not exposed found elevations in microglial and astroglial activation in regions sampled, including striatum[38]. **These studies argue that gliosis may occur after COVID-19, particularly after severe COVID-19, but they do not specifically sample COVID-DNP.**

1.3 Our PET Imaging Study of Gliosis Marker TSPO in COVID-DNP Demonstrates Prominent Involvement of Ventral Striatum and Dorsal Putamen

To our knowledge there are no published studies investigating gliosis in COVID-DNP. In our recently completed study of 20 antidepressant free COVID-DNP and 20 healthy controls, we discovered elevated translocator protein distribution volume, TSPO V_T, a marker of gliosis throughout the key brain regions assessed (ventral striatum, dorsal putamen, hippocampus, anterior cingulate cortex, prefrontal cortex, repeated measures ANOVA (region repeated measure, effect of group, $F_{1,37}=4.3$, $p=0.04$)). Greatest elevations in TSPO V_T were in ventral striatum (22%) and dorsal putamen (20%) (see Fig 1 below). Models of group by region interaction indicate these two regions differ significantly more between COVID-DNP and controls. All initially had mild or moderate severity COVID, reflecting ~85% of wild type cases and 95% of recent strains[2-7].

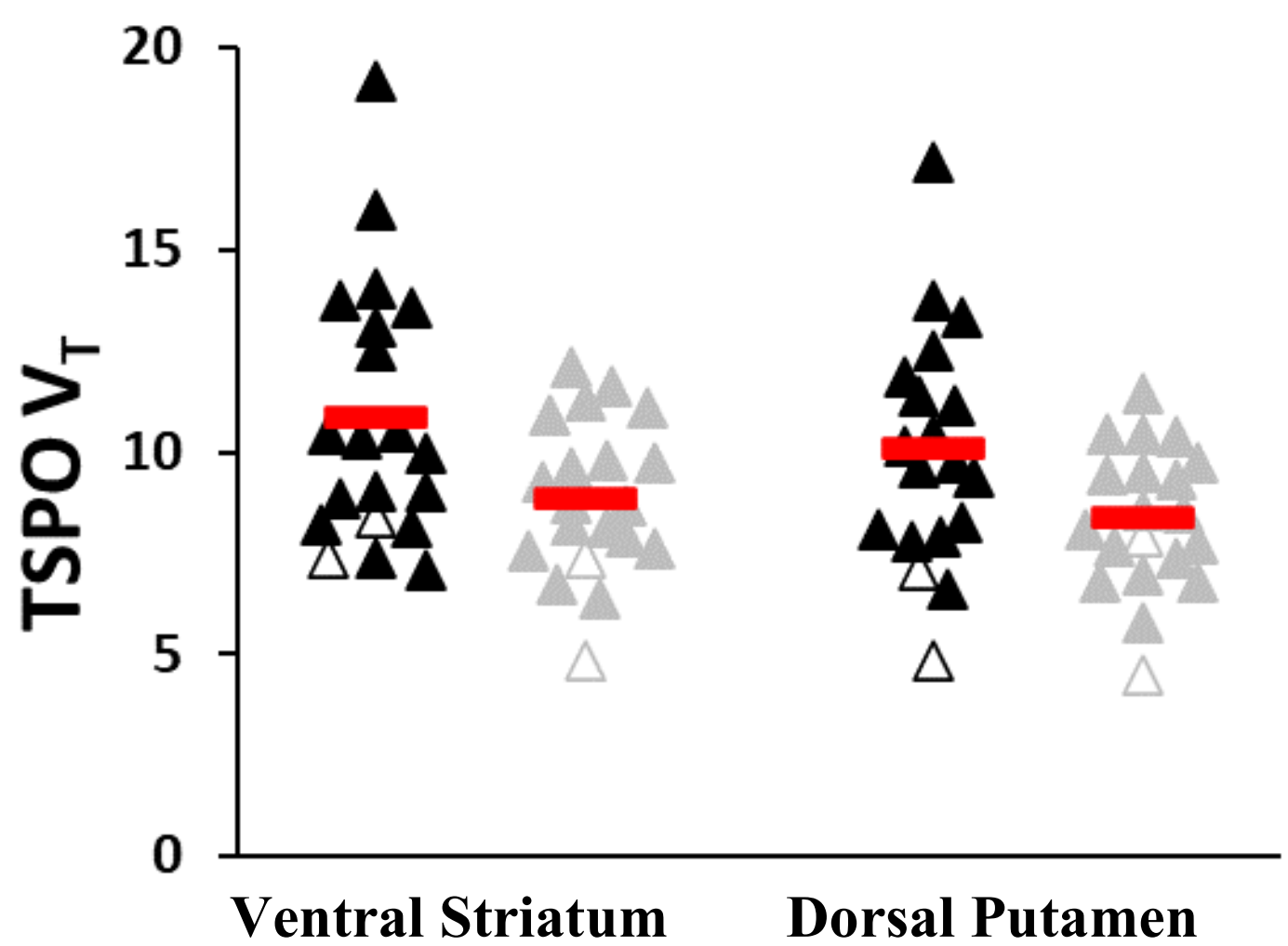

Fig 1.COVID-DNP (HAB ▲ or MAB △) **and Healthy** (HAB ▲ or MAB △). HAB is high affinity and MAB mixed affinity binder of rs6971 genotype, a SNP affecting radiotracer binding to TSPO.

Ventral Striatum TSPO V_T 22% greater in COVID-DNP
ANOVA, $F_{1,37}$=6.2 p=0.018

Dorsal Putamen TSPO V_T 20% greater in COVID-DNP
ANOVA, $F_{1,37}$=6.4 p=0.016
Analysis of variance, ANOVA, evaluating group (patient vs control). Model included rs6971 genotype as separate factor.

The most plausible explanation for greater TSPO V_T in ventral striatum and dorsal putamen in COVID-DNP is gliosis (activated microglia and/or astroglia)[39]. Manuscript in preparation, sample of 20 patients vs.20 controls is considered strong evidence with such methods, as we have 4 papers in JAMA Psychiatry (impact factor 25) with such a sample size[40-43].

1.4 Potential Pathological Effects of Gliosis in COVID-DNP Measurable With PET

In Gliosis, Microglia and Astroglia May Participate in Synaptic Pruning: Activated microglia and astroglia may intervene with their extensions to displace neuronal dendrites and remove synapses, lowering presynaptic density (glia with this role sometimes termed "the synaptic stripper")[44-47]. This may be healthy during neurodevelopment and maintenance of normal function but when excessive it is viewed as pathological[44-47]. This process is proposed to cause cognitive impairment associated with unhealthy aging and neurodegenerative disease[44-48] and it has been proposed that activated microglia[49] contribute to the synaptic loss in MDE of major depressive disorder (MDD)[50, 51].

Loss of SV2A Protein May Be Applied as a Marker for Loss of Synapses: Proteins located in the membrane of synaptic vesicles are often applied as markers of synaptic density. SV2A is such a marker and is located in presynaptic vesicles throughout grey matter regions of the brain [52]. SV2A colocalizes with a high proportion of synapses (82% of synapses with VGlut-1 and 96% of GABAergic synapses;[53]) and its level is highly correlated with a standard postmortem marker of presynaptic density, SYN density (r=0.96)[54]. *PET imaging for SV2A proteins is highly advanced so binding to this marker can be quantitated in vivo (section 3.2)[55, 56].*

Gliosis in the striatum is implicated in causing loss of dopamine releasing neurons [22, 57]. Gliosis is implicated in damaging dopaminergic nerve terminals through multiple mechanisms such as generation of reactive oxygen species leading to toxicity and/or synaptic pruning[58-60]. Empirically, induction of gliosis in the striatum such as through lipopolysaccharide

administration, is associated with loss of dopamine releasing neurons; as shown through reduced VMAT2 density[58-60].

VMAT2 is well established as a marker of dopamine neuron loss. VMAT2, is a protein in the synaptic vesicles of monoamine releasing neurons and a preferred index of dopamine releasing neurons in striatum[61, 62]. About 95% of the VMAT2 binding in the striatum is attributed to dopamine releasing neurons[61, 62]. Administration of dopamine neuron toxin 1-methyl-4-phenyl-1,2,3,6-tetrahydropyridine (MPTP) is associated with loss of VMAT2 density in rodents and primates[63, 64]. Furthermore, loss of ventral striatum and/or dorsal putamen dopamine nerve terminals in humans with Parkinson's disease, progressive supranuclear palsy, and multiple system atrophy is detectable in postmortem study and/or VMAT2 PET imaging[65-69]. *PET imaging for VMAT2 is highly advanced so binding is highly quantifiable in vivo (section 3.2)*[55, 56].

1.5 Loss of Dopamine Releasing Neurons in Ventral Striatum and Dorsal Putamen as a Transdiagnostic Pathological Mechanism for Symptoms of COVID-DNP

Finger tapping speed is a well established, highly reliable measure of motor speed. This test is regularly applied in Dr. Meyer's laboratory as part of a battery of neuropsychological tests to assess certain types of symptoms[70, 71].

Performance on this test may be converted into T-scores corrected for age and sex.

<u>In our previous sample of unmedicated COVID-DNP described in section 1.3, there was a strong correlation between finger tapping speed and TSPO V_T in a set of 19 cases.</u> See Fig. 2.

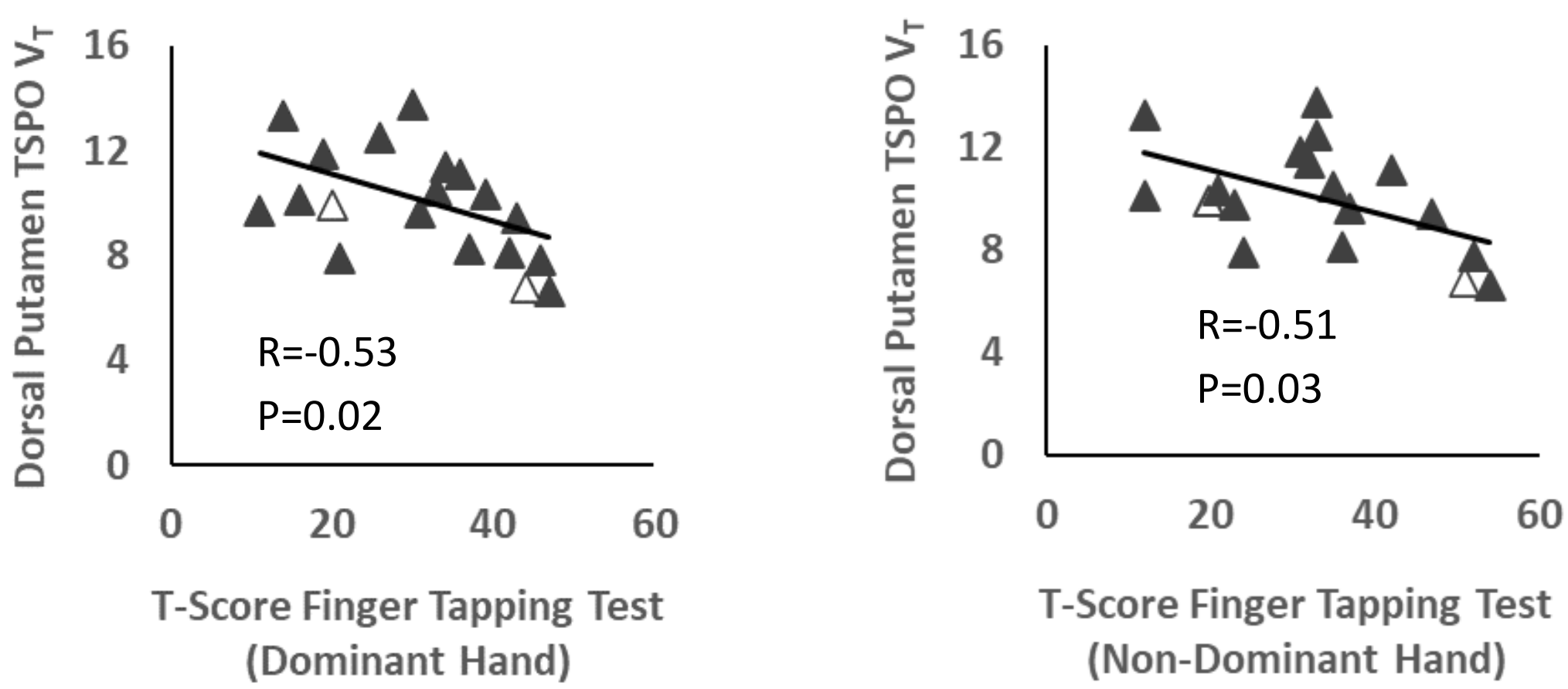

Fig. 2 Significant correlations between age and sex corrected T-score for finger tapping speed and TSPO V_T in dorsal putamen in COVID-DNP. HAB (▲) is high affinity; MAB (Δ) mixed affinity of rs6971 genotype which affects radiotracer binding to TSPO. MAB scores were corrected in this figure by a scale factor of 1.4 (near identical results with alternative linear regression approach to correction of adding difference of MAB effect).

A plausible explanation for figure 2 above is that gliosis led to loss of dopamine releasing neurons (as described in section 1.4) and that loss of dopamine releasing neurons in dorsal

striatum led to symptoms of motor retardation (mechanism described further in section 1.5). Also note in the above figure that motor retardation is considerable in COVID-DNP with the mean T-score being ~25th percentile[22, 24, 72].

Symptoms of anhedonia, motor retardation and low energy (one component of which is low motivation[73]) are prominent in COVID-DNP [22, 24, 72]. It is well established that the ventral striatum, particularly dopamine releasing neurons participate in anticipating, predicting, valuing reward[74-76]. Dopamine releasing neurons participating in these functions extend into the dorsal putamen[74]. The dorsal putamen participates in maintaining normal movement speed and motivation[76-79]. **Hence the functions of the ventral and dorsal striatum, if dysregulated, may contribute to several important symptoms of COVID-DNP, and, as shown in section 1.4, the ventral striatum and dorsal putamen had the greatest elevation in TSPO V_T. Such symptoms are transdiagnostic.** In human diseases that have loss of dopamine releasing neurons extending into ventral striatum are associated with anhedonia such as in Parkinson's Disease[80-82], and 1-methyl-4-phenyl-1,2,3,6-tetrahydropyridine (MPTP) toxicity[83-85]. Similarly, human diseases that reduce dopamine releasing neurons in dorsal striatum are associated with motor retardation and amotivation such as in Parkinson's Disease[80-82], progressive supranuclear palsy[86, 87], multiple system atrophy[86-88] and MPTP toxicity[83-85]. **Anhedonia, motor slowing and low motivation symptoms correspond to function of Research Domain Criteria (RDOC) systems** (and specific constructs) respectively of positive valence systems (reward responsiveness), sensorimotor (motor execution) and arousal (motivated behavior).

1.6 ACE2 Expression is High in Ventral Tegmental Area and Substantia Nigra, Which Might Lead to Loss of Synapses and Gliosis in Striatum

Since SARS-CoV-2 is taken up into cells via ACE2 receptors, it has been proposed that brain regions with relatively greater expression of this receptor may be more vulnerable. Within the brain **two regions with relatively higher levels compared to other brain regions are the substantia nigra[89] and ventral tegmental area[90].** It is acknowledged that some hypothalamic nuclei also have relatively higher levels (paraventricular, superoptic, mamillary[90]), followed by much lower level, with decreasing expression, hippocampus[89], caudate, anterior cingulate cortex and prefrontal cortex, caudate, other cortex regions[89].

Given ventral tegmental area (VTA) and substantia nigra (SN) projections to the ventral striatum and dorsal putamen respectively, lesions to the VTA and SN are known to cause gliosis in the ventral and dorsal putamen[91-93]. 6-OHDA lesions in the VTA and/or SN in rodents may elicit elevations in TSPO binding in striatum for a couple of months, as well as long lasting reductions in VMAT2 and reductions in SV2A level[91-93].

1.7 Overview Theory of Anhedonia, Motor Slowing, Low Energy and Synaptic Loss in COVID-DNP (Occurring After Mild to Moderate COVID)

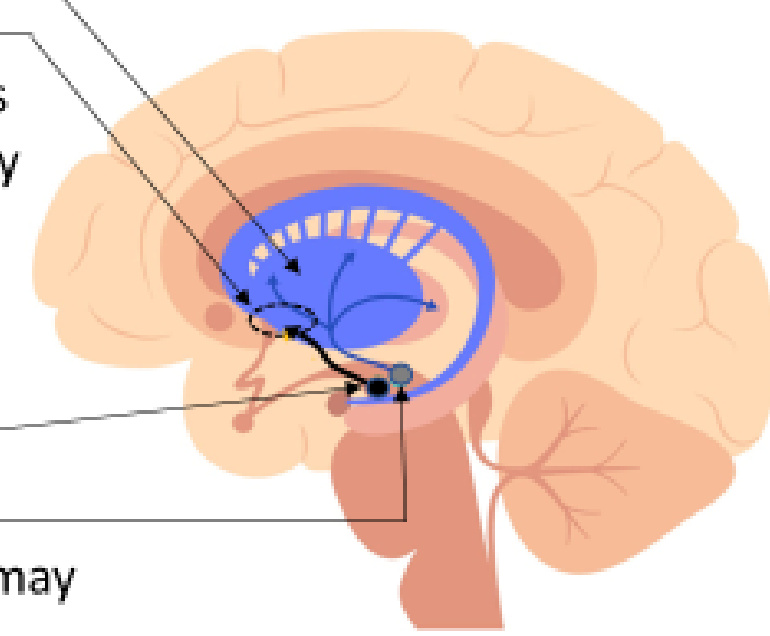

1.8 Translational Outcomes

Should VMAT2 and/or SV2A binding be reduced, this would justify clinical trials of specific therapeutics for COVID-DNP. This can be done quickly because medications are already available for other indications and can be repurposed; and there is evidence of target engagement for many of them which is needed to justify clinical trials. *Proposed medications* and *evidence of target engagement*, are presented. **Loss of Striatal VMAT2** implies loss of dopamine releasing synapses, therefore therapeutics raising dopamine would be anticipated to be helpful. Monoamine oxidase B and A (MAO-B, MAO-A) metabolize dopamine, and inhibiting the dopamine transporter elevates magnitude of synaptic dopamine release so the following medications are proposed, but others like COMT inhibitors, could be considered.

Selective MAO-B Inhibitors: *The PI is studying MAO-B occupancy of* **rasagiline** *using [^{11}C]SL25.1188 PET*[40, 94] *and at a therapeutic dose not requiring dietary restrictions for tyramine sensitivity, it has a sustained occupancy of 85%; a finding consistent with a previous semi-quantitative PET study*[95]. Also, rasagiline is well tolerated[96] and had neuroprotective effects preserving dopamine releasing neurons in a rodent model of Parkinson's disease (PD)[97]. Rasagiline is available in the United States for PD. While most MAO-B inhibitors were developed for PD, EMSAM, a patch form of l-deprenyl was developed as an antidepressant and is available in United States[98].

Selective MAO-A Inhibitors: Moclobemide is a selective MAO-A inhibitor[99] and *the PI published the only MAO-A occupancy of moclobemide which exceeds 75% at clinical dosage not requiring dietary restrictions for tyramine sensitivity*[100]. This medication is not available in the United States but could be developed for the US market.

Dopamine Reuptake Inhibitor: Bupropion is a commonly used antidepressant available in the United States. A key mechanism of its action is through binding to the dopamine transporter, which based on collective review of occupancy studies has an occupancy about 15%[101] (*The PI was the first to study this*[102]). It is generally believed that a modest occupancy of the DAT still has substantial potency[102, 103], in contrast to the need for higher occupancy of other antidepressant targets like the serotonin transporter[104].

Loss of SV2A implies a generalized loss of synapses in striatum so therapeutics to generally raise presynaptic density could be considered. This includes medications approved for MDE of major depressive disorder (MDD) like **esketamine**[105, 106], medications approved for other use like **ketamine**[107]; and medications in development in clinical trials in humans such as AMPA receptor potentiators in development like **TAK-653**[108, 109] (in phase 2 clinical trial[110]); and TORC-1 signalling agents like **NV-5138**[111](in phase 2 clinical trial[112]). In MDE of MDD, target engagement of ketamine was supported by a [^{11}C]UCB-J PET study reporting elevations in SV2A V_T post-treatment in cases with pre-existing low SV2A V_T.[113]

2. STUDY OBJECTIVES

2.1 Main Goal/Research Aims
(1) We will determine if VMAT2 BP_{ND} is reduced in the ventral striatum and dorsal putamen in COVID-DNP.

(2) We will determine if SV2A V_T is reduced in the ventral striatum and dorsal putamen in COVID-DNP.

(3) We will assess the relationship between measures of anhedonia (anticipating/predicting /valuing reward) with VMAT2 BP_{ND} and SV2A V_T in ventral striatum in COVID-DNP.

(4) We will assess the relationship between measures of motor speed, cognitive processing speed and motivation with VMAT2 BP_{ND} and SV2A V_T in dorsal putamen in COVID-DNP.

Exploratory aims will include (i) assessing the relationship between VMAT2 BP_{ND} and SV2A V_T in ventral striatum and in dorsal putamen; (ii) comparing VMAT2 BP_{ND} between groups in other brain regions including caudate and midbrain; and (iii) comparing SV2A V_T between groups in other brain regions, including hippocampus, prefrontal and anterior cingulate cortex

2.2 Main Hypotheses
(1) VMAT2 BP_{ND} is reduced in the ventral striatum and dorsal putamen in COVID-DNP.

(2) SV2A V_T is reduced in the ventral striatum and dorsal putamen in COVID-DNP.

(3) Greater anhedonia will be correlated with lower VMAT2 BP_{ND} and SV2A V_T in ventral striatum in COVID-DNP.

(4) Slower motor speed, cognitive processing speed and reduced motivation will be correlated with lower VMAT2 BP_{ND} and SV2A V_T in dorsal putamen in COVID-DNP.

Exploratory Hypotheses are (i) VMAT2 BP_{ND} and SV2A V_T will be positively correlated in ventral striatum and in dorsal putamen; (ii) VMAT2 BP_{ND} will be lower in other brain regions in COVID-DNP such as caudate and midbrain (iii) SV2A V_T will be reduced in the hippocampus, (trend to greater TSPO V_T in hippocampus in COVID-DNP in previous dataset).

3. METHODS

3.1 Study Design

Design Structure: Participants undergo pre-screening, screening, two different PET scans (one for VMAT2 (using radioligand (+)[^{11}C]DTBZ) and one for SV2A (using radioligand [^{18}F]SDM8 (often called [^{18}F]SynVesT-1 in the literature) and MRI scanning. If a participant is unable or unwilling to complete the SV2A PET scan, or the SV2A PET scan is not available, it will not be rescheduled and remaining study procedures will be completed as described. This is a cross-sectional, observational study.

Overview of Visits and Expected Study Duration: Participants will undergo one telephone pre-screening visit, one Study Q&A discussion (unless the participant wishes to have the consent process completely done in-person), one in-person screening visit, and a maximum of five additional visits to complete neuroimaging and psychometric study procedures. Note that the informed consent procedure (and signing of the consent form if individuals choose to participate) will occur during the in-person screening visit. The duration between each PET scan is intended to be less than 4 weeks. The maximum study duration is 4 months but is typically expected to be less than 2 months. The Schedule of Events outlines the study visits for Groups 1 and 2 (Table 5.1) Groups are defined in Section 3.2. All in-person study procedures will be conducted at CAMH, 250 College Street, Toronto.

Number of Sites: Single site study, with procedures taking place at CAMH. Scanning will take place at the CAMH Brain Health Imaging Centre.

3.2 Participants

Overview of Participant Groups

Group 1: Recovered from mild to moderate COVID-19 respiratory symptoms with subsequent onset of COVID-DNP (n=45 completers)

Group 2: Healthy controls (n=45 completers)

Previously or Concurrently Recruited Data for Group 2: Up to 45 additional scan data from [^{18}F]SDM8 ([^{18}F] SynVesT-1)PET scans in which CAMH REB approval has been given for use in common databases for general use, may be added to Group 2, provided participants meet the study criteria for healthy control participants (see Section 4) are of good health, have no history of psychiatric disorders as found on the Research version of Structured Clinical Interview for DSM-5[114]. Up to 45 additional scan data from (+)[^{11}C]DTBZ PET scans in which CAMH REB approval has been given for use in common databases for general use, may be added to Group 2, provided participants meet study criteria for healthy control participants (see Section 4) are of good health, have no history of psychiatric disorders as found on the Research version of Structured Clinical Interview for DSM-5[114] or the Structured Clinical Interview for DSM IV[115]. Participant data from healthy control participants are also anticipated to come from previous

healthy study participants who have received (+)[^{11}C]DTBZ PET scans (Kish lab studies REB# 001/2006, 017/2014 and Boileau lab study REB# 002/2006) who are of good health, have no history of psychiatric disorders as found on the Research version of Structured Clinical Interview for DSM-5[114] or the Structured Clinical Interview for DSM IV. A waiver for the requirement of informed consent for the secondary use of information is being applied.

4. PARTICIPANT SELECTION AND WITHDRAWAL

4.1 Inclusion/Exclusion Criteria

This study will be conducted in line with local and international ethical research guidelines on obtaining informed consent from human research participants. Participant recruitment will not commence until full approvals have been granted from Health Canada and CAMH REB. Only study participants who meet the below defined inclusion/exclusion criteria will be enrolled in the study.

Group 1: Recovered from mild to moderate COVID-19 respiratory symptoms with subsequent onset of COVID-DNP

Inclusion criteria:

i. New onset MDE within 3 months after mild or moderate COVID-19, with MDE verified by the research version SCID for DSM 5[114]. See "Positive COVID-19 diagnosis" listed after inclusion/exclusion criteria for a description of how this is determined.

ii. Age 18 to 75

iii. Good general physical health with no active medical conditions based on self-report (except migraine or PASC)

Exclusion criteria:

i. Use of antidepressants in the previous month (6 weeks for fluoxetine)

ii. Use of stimulant medication affecting dopamine release in the previous month

iii. Use of antipsychotics in the previous month

iv. History of neurological disease (except migraine, and PASC) based on self-report

v. Use of medications or herbal products or natural health products with central nervous system effects in past 2 weeks based on self-report

vi. Presence of cigarette smoking in the past two months, based on self-report

vii. Lifetime diagnosis of severe Substance or Alcohol Use Disorder, or diagnosis of mild to moderate Substance or Alcohol Use Disorder in the past two years, based on self-report and

verified by the Research Version of Structured Clinical Interview for Diagnostic and Statistical Manual-5 (SCID-5-RV)

viii. Use of recreational drugs, including marijuana, in the past two months, based on self-report and verified by the Research Version of Structured Clinical Interview for Diagnostic and Statistical Manual-5 (SCID-5-RV)

ix. Positive urine drug or cotinine screen at any timepoint during the study

x. History of psychotic symptoms secondary to schizophrenia, schizophreniform, bipolar disorder, or brief psychotic disorder prior to COVID-19, based on self-report and verified by the Research Version of Structured Clinical Interview for Diagnostic and Statistical Manual-5 (SCID-5-RV)

xi. Currently pregnant, based on self-report or positive pregnancy test at any timepoint during the study, in females (in our PET Centre females up to 65 years of age are given a urine pregnancy test prior to every PET scan)

xii. Breastfeeding (for females)

xi. Current disorders of coagulation, blood or ongoing use of anticoagulant medication, based on self-report

xii. Claustrophobia, based on self-report

xiii. Weight over 400lbs and height over 7ft (requirements for fitting in the scanners and hospital gowns)

xiv. History of undergoing a number of PET scans that will lead participants to exceed the annual (20mSv) / lifetime (8 PET scans) radiation limit by completing this study

xv. Presence of metal implant, object or electrical devices that are contraindicated for MRI, based on self-report

xvi. Severe allergic reaction to alcohol

Group 2: Healthy controls

<u>Inclusion criteria:</u>

i. Age 18 to 75

ii. Good physical health with no current medical conditions (except migraine)

iii. Good psychiatric health including negative screen on the research version SCID for DSM5[114]

Exclusion criteria:

i. History of PASC (based on self-report on structured symptoms questionnaires)

ii. History of moderate or severe acute COVID-19

iii. History of neurological disease (except migraine, and in COVID-DNP, PASC) based on self-report

iv. History of psychotropic medication treatment

v. Use of medications or herbal products or natural health products with central nervous system effects in past 2 weeks based on self-report

vi. Presence of cigarette smoking in the past two months, based on self-report

vii. Lifetime diagnosis of severe Substance or Alcohol Use Disorder, or diagnosis of mild to moderate Substance or Alcohol Use Disorder in the past two years, based on self-report and verified by the Research Version of Structured Clinical Interview for Diagnostic and Statistical Manual-5 (SCID-5-RV)

viii. Use of recreational drugs, including marijuana, in the past two months, based on self-report and verified by the Research Version of Structured Clinical Interview for Diagnostic and Statistical Manual-5 (SCID-5-RV)

ix. Positive urine drug or cotinine screen at any timepoint during the study

x. History of psychotic symptoms secondary to schizophrenia, schizophreniform, bipolar disorder, or brief psychotic disorder prior to COVID-19, based on self-report and verified by the Research Version of Structured Clinical Interview for Diagnostic and Statistical Manual-5 (SCID-5-RV)

xi. Currently pregnant, based on self-report or positive pregnancy test at any timepoint during the study, in females (in our PET Centre females up to 65 years of age are given a urine pregnancy test prior to every PET scan)

xii. Breastfeeding (for females)

xi. Current disorders of coagulation, blood or ongoing use of anticoagulant medication, based on self-report

xii. Claustrophobia, based on self-report

xiii. Presence of metal implant, object or electrical devices that are contraindicated for MRI, based on self-report

xiii. Weight over 400lbs and height over 7ft (requirements for fitting in the scanners and hospital gowns)

xiv. History of undergoing a number of PET scans that will lead participants to exceed the annual (20mSv) / lifetime (8 PET scans) radiation limit by completing this study

xv. Severe allergic reaction to alcohol

Positive COVID-19 diagnosis is a history of the presence of symptoms and a past positive polymerase chain reaction test from either a nasopharyngeal swab, nasal, or throat swab[116], or another validated test appropriate to illness state, such as an antibody-based test in an unvaccinated state or a positive antigen test. Preferred documentation includes provision by the participant of a copy/screenshot of the results from the online portal used in Ontario or positive antigen test, or a doctor's note, or a copy of a medical record. The latter will be redacted by standard methods at CAMH to be de-identified. However, given that the results of COVID-19 testing on the online portal in Ontario may not be available after a time window of a few months, and that many people do not keep screenshots of their antigen test results, self-report of a positive test (listed above) will also be accepted. **For severity**, we apply the definitions of Health Canada[117] and the World Health Organization[118]. Moderate COVID-19 is the presence of clinical symptoms of pneumonia but not severe enough to require ongoing use of supplementary oxygen; and mild COVID-19 is symptoms but no evidence of pneumonia or hypoxia. <u>Numbers for each group assume 10% dropout rate. A similar number of males and females will be recruited for each group.</u>

4.2 Participant Withdrawal

Study participants may withdraw consent at any time without explanation. Should a participant withdraw consent after being enrolled in the study, study staff will attempt to contact the participant to resolve any outstanding emergent adverse events at the early termination visit. Following the early termination visit, participants will not be asked to complete any further study procedures, nor will additional data be collected. Study data collected prior to participant withdrawal will be retained unless the participant requests complete withdrawal of study data. The participant may also request that their DNA sample and/or blood samples be destroyed at any time. The Principal Investigator/Qualified Investigator (PI/QI) will ensure that destruction of biological samples be completed within a reasonable timeframe.

The PI/QI may choose to withdraw study participants who meet any of the below criteria. Participants with emergent adverse events (AEs) who are withdrawn by the PI/QI will be followed until the adverse events are resolved.

PI/QI withdrawal criteria:

i. Any change in medical or psychiatric status during enrolment that would meet exclusion criteria listed in Section 4.1

ii. Participant non-compliance of study procedures outlined in the Protocol and Informed Consent Form

iii. Participant experiences a serious adverse event (SAE) during study participation

iv. If, at the discretion of the PI/QI, it is not in the best interest of a participant to undergo study procedures outlined in the Protocol and Informed Consent Form

4.3 Participant Recruitment

Participants are recruited by posting advertisements at CAMH, local newspapers, online (including the CAMH Research Registry) and within the Greater Toronto Area. The present study will be advertised in the form of posters and brochures at local medical and psychiatric clinics, including at hospitals (if regulations at such sites permit), community centres, and around the community. Participants will also be recruited through advertisement or through the outpatient services at General & Health Systems Psychiatry at CAMH. Additionally, the study will be advertised through print in newspapers and online. Potential participants who are interested can use the contact information featured on these advertisements to contact the CAMH research personnel involved in the study. Upon calling or emailing a study member, they will be further informed about the study and the CAMH research personnel shall obtain informed consent as appropriate.

More specifically, use of study advertisements, flyers and brochures may include the following:

- Use of the CAMH Research Registry, CAMH member database and the CAMH website such as camh.ca
    - Links to the posting of the study here will be incorporated into other online postings
- Advertisements/flyers/brochures posted in the community (including community centres, university campuses, drugstores, community boards, public transportation, religious centres, coffee shops, and health and wellness organizations)
- Advertisements/flyers in online and print magazines, newsletters, and newspapers
- Advertisements/flyers/brochures and presentations at relevant conventions/shows
- Online/website advertisements on social media including those of community, and health and wellness organizations (including their Facebook, Twitter, Instagram, and blog accounts); websites Kijiji and Craigslist; and search engines (like Google and Yahoo)
- Advertisements/flyers/brochures posted in clinics
- Advertisements/flyers/brochures posted in hospitals

The study will also be advertised through sponsored content articles that will be published in local newspapers. These articles will also be made available on the respective publisher websites and will be promoted on their social media accounts (such as Facebook and Instagram).

One of our additional routes of recruitment will be to employ the Clinical Engagement and Research Recruitment (CLEARR) model. The CLEARR designated Research Coordinator under the supervision of a team recruiting clinician and accountable to the Manager of Research Practice will identify (flag) potential participants and notify the client's clinician about the client's eligibility to participate in this research study. The client's clinician will then ask their client if they would be willing to meet with a study team member about participating in this study. Only with a clients' agreement will they be approached.

4.4 Participant Compensation

Participants will be compensated in cash, on a prorated basis, as outlined below. If the end of study visit is done in person, they will be paid in person. If the end of study visit is done virtually, they will be paid by mail. Participants may be compensated in cash for lunch if the study visit occurs over the lunch period and for transportation home after the removal of arterial lines, following the submission of receipts.

Visit	Compensation	Time	Description
Visit 1: Screening	$90	4 hours	Informed consent process, eligibility review, demographics, medical and psychiatric history, medications, height and weight, clinical interviews, urine sample, MRI screening form and symptom review.
Visit 2: VMAT2 PET scan	$165	6 hours	Cognitive and clinical tests and questionnaires, urine and blood samples, PET scan and symptom review.
Visit 3: SV2A PET scan	$255	6 hours	Cognitive and clinical tests and questionnaires, urine and blood samples, PET scan and symptom review.
Visit 4: MRI day	$75	2 hours	Cognitive and clinical tests and questionnaires, MRI scan and symptom review.
Visit 5: End of study	$10	30 minutes	Symptom review and reimbursement
Total	$595	18.5 hours	

5. STUDY PROCEDURES

All in-person study procedures will be conducted at CAMH, either at 250 College Street, Toronto or 1001 Queen St. During all the in-person visits, institutional and provincial guidelines for COVID-19 prevention, such as use of personal protective equipment (PPE), will be followed for the safety of both study participants and research personnel.

5.1 Schedule of Assessments

For Groups 1 and 2, all study visits will be completed within a span of 16 weeks, although it is expected that the typical timeframe will be within 8 weeks. The Schedule of Assessments in Table 5.1 outlines the study procedures for participants in Groups 1 and 2. Of note, some study visits may be combined into a single visit (day) or conducted over an additional visit depending on the participant's preference, timing and staffing. Flexibility of study visits is noted in the Schedule of Assessments tables below.

At the discretion of the study's QI, confirmatory analysis and the repeat of assessments will be performed in a timely manner. In addition, at the discretion of the study's QI, the timing of events will be adjusted to accommodate the participant's needs. Also, if there is a lack of availability of PET scans, only one PET scan may be done.

Table 5.1. Schedule of Assessments for Groups 1 and 2

	Phone pre-screen visit	Study Q&A Discussion[1]	Visit 1: Screening	Visit 2: VMAT2 PET scan[2]	Visit 3: SV2A PET scan[2]	Visit 4: MRI scan[2]	Visit 5: End of study visit
**Enrolment procedures**							
Telephone pre-screening script	X						
Participant pre-screening form	X						
Informed consent process		X	X				
Inclusion/exclusion criteria			X	X[3]	X[3]	X[3]	
**Demographics and health status**							
Demographics, medical and psychiatric history, medications			X				
BMI measurement			X				
Ethnicity, questionnaire			X				
COVID-19 questionnaire			X				
**Clinical and cognitive assessments**							
SCID-5-RV*			X				
SCID-5-PD*				X[3]	X[3]	X[3]	
Anhedonia assessments[5]				X[3]	X[3]	X[3]	
Psychomotor assessments[5]				X[3]	X[3]	X[3]	
Amotivation assessments[5]				X[3]	X[3]	X[3]	
Symptom inventories[5]				X[3]	X[3]	X[3]	
Neurocognitive battery[5]				X[3]	X[3]	X[3]	
**Biological specimen collection**							
Urine drug & cotinine screen			X	X	X		
Urine pregnancy test			X	X	X		
Blood markers & proteins				X	X		
**Neuroimaging**							
MRI screening form			X	X[4]	X[4]	X[4]	
[^{11}C]-(+)-DTBZ PET scan				X			
[^{18}F]SDM8/SynVesT1 PET scan					X		
GE Discovery 3T MRI scan				X[3]	X[3]	X[3]	
**Safety**							
Baseline symptom questionnaire			X				
Adverse events review				X	X[3]		X

**Body Mass Index (BMI); Structured Clinical Interview for Diagnostic and Statistical Manual-5 (SCID-5-RV); Structured Clinical Interview for Personality Disorders for Diagnostic and Statistical Manual-5 (SCID-5-PD, subsections for Antisocial and Borderline Personality Disorder only)*

[1]*Study Q&A discussion is optional*

[2]*Participants may complete Visit 3 procedures before Visit 2.*

[3]*Participants have the option of completing assessments and the MRI scan across Visits 2, 3, and 4 depending on their preference and scheduling availability. If participants find it difficult to complete all the assessments within the specific visits, they may have additional 1-2 visits to complete them. MRI may be done prior to PET scanning depending on availability.*

[4]*Review form for changes since Visit 1. Will occur on day of MRI scan.*

[5]*As listed in sections 6.2 and 6.3*

5.2 Study Visits

5.21 Screening Procedures

Telephone Pre-screening Visit

The pre-screening procedure will take place over the phone. Research staff will go over the Telephone Pre-Screening Script and the PET Research Participant Pre-Screening Form with the participant. The pre-screening visit should take approximately fifteen minutes to complete.

If the participant successfully passes the pre-screening visit, then research staff will ask the participant if they may send them a copy of the Informed Consent Form (ICF) to consider prior to the first in-person visit. Research staff will ask participants if they would like the ICF emailed to them and will offer to set up a time to discuss this material via WebEx or phone, prior to the first in person visit.

Study Q&A Discussion (optional)

During the optional Study Q&A discussion, participants will meet via WebEx or telephone to go over the ICF in detail and ask any questions they may have. This conversation is not meant to replace the informed consent procedure performed at the beginning of the first in-person visit, but to provide potential participants with detailed study information prior to their first in-person visit should they be interested. If potential participants are interested in participating following the Study Q&A discussion, they will be invited to attend the in-person screening visit.

Visit 1: Screening

Trained research staff will call/email participants two to three days prior to Visit 1 to remind them of their appointment, with up to four attempts should no answer occur. Prior to initiating study procedures or collecting data, research staff will obtain written informed consent from study participants. Research staff will review the inclusion/exclusion criteria with study participants and document study eligibility. Participants will be asked questions about their age, sex, ethnicity, level of education, medical and psychiatric history, medications, and COVID-19 history using a structured questionnaire. Height and weight measurements will be collected to determine body mass index (BMI), using imperial or metric units but not a mixture of both. Participants will also complete:

1. Research Version of Structured Clinical Interview for Diagnostic and Statistical Manual-5 (SCID-5-RV)
2. Urine drug screen (including test for presence of cotinine)
3. Pregnancy test (in females up to 65 years of age)
4. CAMH MRI screening form
5. Baseline symptom questionnaire

Drug, cotinine and pregnancy exclusion criteria will be analyzed either by laboratory testing or by a urine dipstick test.

This visit should last approximately 4 hours. Enrollment is confirmed once the urine screen results have been obtained and reviewed by the QI. Participants are contacted via phone and/or email (based on indicated preference) to inform them of their eligibility and to schedule a date for the PET scan. An email confirmation of the PET scan date will be sent.

5.22 Procedures After Screening

Visit 2: VMAT2 PET Scan

Trained research staff will call/email participants two to three days prior to Visit 2 to remind them of the appointment, with up to four attempts should no answer occur. There are several clinical assessments and a neurocognitive battery as outlined in sections 6.2 and 6.3, that participants may complete across Visits 2, 3, and 4 depending on timing and participant preference.

Participants will provide a urine sample to screen for recreational drugs (and marijuana), cotinine and pregnancy (in females up to 65 years of age) prior to the PET scan. Drug, cotinine and pregnancy exclusion criteria will be assessed using a urine dipstick test for same-day results. Only participants who screen negative for all three exclusion criteria (or two for males) will undergo the PET scan. The PET scan itself (after radiotracer administration) is expected to last up to 2 hours. Participants will undergo a blood draw intravenously prior or during the PET scan to collect samples for proteins and markers. Participants will be asked to complete a review of adverse events at the end of the visit to monitor for safety. This visit should last approximately 6 hours.

Visit 3: SV2A PET Scan

This scan is optional, as participants may choose to not have this scan as some may find two scans onerous.

Trained research staff will call/email participants two to three days prior to Visit 3 to remind them of the appointment, with up to four attempts should no answer occur. Any clinical or neurocognitive assessments, as outlined in sections 6.2 and 6.3, not completed at Visit 2 can be administered at Visit 3.

Participants will provide a urine sample to screen for recreational drugs (and marijuana), cotinine and pregnancy (in females up to 65 years of age) prior to the PET scan. Drug, cotinine and pregnancy exclusion criteria will be assessed using a urine dipstick test for same-day results. Only participants who screen negative for all three exclusion criteria (or two for males) will undergo the PET scan. The PET scan itself (after radiotracer administration) is expected to last up to 2 hours. Participants will have an arterial blood line placed and undergo a blood draw during the PET scan to collect samples for the radiotracer. Participants will undergo a blood draw intravenously prior or during the PET scan to collect samples for proteins and markers but these samples will be taken from the arterial blood line if the venous sample cannot be obtained. Participants will be asked to complete a review of adverse events at the end of the visit to monitor for safety. This visit should last approximately 6 hours.

Visit 4: MRI Scan

Trained research staff will call/email participants two to three days prior to Visit 4 to remind them of the appointment, with up to four attempts should no answer occur. Study staff will review the MRI Screening Form with participants to ensure that there have been no changes to their eligibility since screening. Any remaining clinical or neurocognitive assessments that were not administered at Visit 2 and 3 will be completed. Participants will undergo a GE Discovery 3T MRI scan that will last approximately 1 hour. Following the MRI scan, participants will be asked to complete a review of adverse events at the end of the visit to monitor for safety. This visit should last approximately 2 hours.

Visit 5: End of Study Visit

Trained research staff will call/email participants two to three days prior to Visit 5 to remind them of the appointment, with up to four attempts should no answer occur. Participants will complete a final review of adverse events at least one day after Visit 4. Any emergent adverse events will be followed-up to resolution. Participants will be reimbursed at the end of study visit, upon study completion or termination. This visit should last approximately 30 minutes. The visit may take place in person; or remotely via WebEx or phone call, depending on participant preference.

6. ASSESSMENTS

6.1 Demographics, Medical and Psychiatric History, Medication, and COVID-19

Demographic data regarding age, sex, ethnicity and level of education will be collected from study participants. All participants will be asked questions about their complete medical, psychiatric, medication, and COVID-19 history through a structured interview. Ethnicity will be collected on a standard form. Female participants will be asked additional questions regarding their last menses, and if they are currently pregnant or breastfeeding. All self-reported responses will be documented in the applicable source documents (SDs) and case report forms (CRFs).

6.2 Clinical Assessments

All study participants will be screened for psychiatric and substance use disorders using two validated, semi-structured clinical interviews. The Research Version of the Structured Clinical Interview for Diagnostic and Statistical Manual-5 (SCID-5-RV) is a broad, clinician-administered interview that screens for 63 psychiatric disorders listed in the DSM-5[114] This assessment can be administered in approximately 90 minutes. To further characterize the sample of participants, the Structured Clinical Interview for Personality Disorders for Diagnostic and Statistical Manual-5 (SCID-5-PD) which is similarly structured and covers ten personality disorders listed in the DSM-5 will also be applied. However, participants will only be assessed for Antisocial and Borderline Personality Disorders, and thus only relevant portions of the SCID-5-PD[119] will be administered. Administration should take approximately 30 minutes. The childhood trauma questionnaire will also be administered[120].

Additionally, the following clinical assessments will be administered to all participants.

<u>Anhedonia:</u>

- Snaith Hamilton Pleasure Scale (SHAPS)[121]
- Anticipatory and Consummatory Interpersonal Pleasure Scale (ACIPS)[122]
- Effort Expenditure for Rewards Task (EEfRT) [123]
- Delayed Discounting Task (DDT)[124]
- Behavioural Activation Scale and Behavioural Inhibition Scale (BAS/BIS)[125]

<u>Psychomotor Retardation:</u>

- Finger Tapping Test
- Comprehensive Trail Making Test
- Stroop Color-Word Interference Test
- Wechsler Digit Symbol Substitution Test

<u>Amotivation:</u>

- The Inventory of Depression and Anxiety Symptoms II (IDAS-II)
- Lassitude scale[126-129]
- Marin Apathy Scale[130]

Symptom Inventories:

- 17-item Hamilton Depression Rating Scale (HDRS-17)
- Beck Depression Inventory (BDI)
- Beck Scale for Suicidal Ideation (BSSI)
- State Trait Anxiety Inventory (STAI)

6.3 Neurocognitive Assessments

In addition to the aforementioned measures, cognitive performance will be evaluated through a standardized, validated neurocognitive battery that measures as a range of domains assessed with the protocol in our previous publications[70, 71], including:

- Cognitive Failures Questionnaire[131, 132]
- Continuous Performance Task (CPT)
- Wisconsin Card Sorting Test (WCST)
- Hopkins Verbal Learning Test-Revised (HVLT-R)
- Brief Visuospatial Memory Test-Revised (BVMT-R)
- Wechsler Test of Adult Reading (WTAR)
- Boston Naming Test (BNT)
- Benton Judgement of Line Orientation (JLO)
- Probabilistic Learning Task (PLT)

It will take approximately 90 minutes to administer the neurocognitive battery.

6.4 Urine Screen

Study participants will submit a urine sample in order to confirm study eligibility. At Visit 1, urine samples will be analyzed for drugs of abuse, cotinine and pregnancy (in females up to 65 years of age) using standardized laboratory processing techniques. On PET scanning days, the urine screen will be performed using a dipstick for quick same-day results.

6.5 [^{11}C]-(+)-DTBZ PET Scanning

PET radiotracer [^{11}C]-(+)-DTBZ, an excellent PET radiotracer for this target has high brain uptake, excellent selectivity, high affinity (~1nM)[133] and high specific binding relative to free and non-specific binding (~2.5 in humans on our HRRT PET scanner[134]). 370MBq of [11C]-(+)-DTBZ (+/-10%) will be given intravenously as a bolus. Scanning time is approximately 90 minutes.It has been modeled in humans and shows very good reversibility[62, 135]. Moreover, it detects loss of dopamine releasing neurons in the striatum after administration of dopaminergic neurotoxins and in diseases with loss of dopamine releasing neurons[67, 136]. Reliability is excellent with mean absolute test-retest measurement of VMAT BP_{ND} <10%.[135] [^{18}F]FE-(+)-DTBZ is another option but defluorination leading to non-specific radioactivity have been raised as an issue for quantitation[137]. [^{18}F]FP(+)-DTBZ would be reasonable with a similar specific binding to free and non-specific binding as [^{11}C]-(+)-DTBZ [134, 138] (compared time activity

curves identical in same non-human primate[137]) but to our knowledge, [^{18}F]FP(+)-DTBZ lacks modelling validation in human brain.

6.6 [^{18}F] SDM8 ([^{18}F] SynVesT-1) PET Scanning

PET scanning for this research project will be completed on our new GE Discovery MI 5-Ring PET/CT scanner (Milwaukee, Wisconsin, USA) and will be conducted in accordance with CAMH PET standardized operating procedures to image SV2A V_T, an index of SV2A density. 185MBq of [^{18}F]SDM8 **(+/-**10%**)** will be given intravenously as a bolus. An arterial line will be required. The arterial line is a thin catheter inserted into the radial artery in the wrist and is used to withdraw arterial blood samples which are used in conjunction with the PET brain image data to calculate the binding measure. This is the standard methodology for PET imaging when there is no brain region available that is validated to be a reference tissue that can be used as a surrogate input function: i.e. devoid of the radiotracer target, and has non-specific binding similar to the regions with specific binding.

While there is a potential region (foramen semiovale (also termed centrum semiovale)) that may be close to meeting reference tissue requirements, the first publication applying a highly similar radioligand ([^{11}C]UCB-J) reported an effect of a major depressive episode itself on this potential reference region that would confound its use[67]. Hence, the current standard in the field is to use arterial blood sampling for investigations in major depressive episodes for SV2A PET radiotracers and for first time studies in new disease conditions, particularly if they often have comorbid major depressive episodes.

Prior to insertion, lidocaine, a local anesthetic, is applied in a small dose to numb the area and to make the insertion of the catheter easier. The arterial line is inserted by a registered respiratory therapist. A low dose scout scan is initially acquired to allow for accurate positioning of the subject for the CT and PET acquisitions. On completion of the scout, and prior to the administration of **[**^{18}F]SDM8, a second low dose CT scan will be acquired which will be used for both scatter and attenuation correction of the PET data. During PET scanning, no more than 150mL of arterial blood will be taken as part of the radiotracer measurements. Scanning time is 120 minutes.

6.7 Additional Blood Sampling with PET Scanning

At each PET scan, additional blood samples will be taken for chemistry, hematology, and peripheral biomarker that would logically be expected to predict VMAT2 and SV2A levels in the brain, gliosis, and mood and anxiety dysregulation analyses. A maximum of 220 mL total will be collected over both PET scans. The breakdown of blood taken is as follows:

- no more than 150 mL is used for radiotracer analysis:
    - 0 mL for VMAT2 scan (no arterial blood sampling)
    - no more than 150 mL for SV2A scan
- 20 mL is used for protein binding analysis to measure binding of the radiotracer to plasma protein (for SV2A scan only)
- 25 mL used for inflammatory marker analysis or other markers implicated in loss of VMAT2 or SV2A (done for each PET scan)

Blood will be collected during each PET scan day and allowed to clot for 30 minutes at room temperature before centrifugation for 10 minutes at 1000 x g (4° C preferred). Serum will be removed and aliquots will be stored in -80° C until processing.

Blood samples will be kept during the study period and beyond for subsequent and potentially pooled analysis; following completion of all analyses these blood samples will be destroyed. Analysis of blood samples will be performed at CAMH and/or with external collaborators. Some samples of blood will be sent out to other specialized laboratories to look at specific biomarkers implicated in VMAT2 and SV2A regulation, inflammation and mood disorders.

All blood samples will be stored and frozen at -80C in a freezer maintained by the PI/QI at CAMH, 250 College or Queen St. sites. The freezer is located in a secure, locked location to protect from unauthorized handling of samples. Blood samples will be labeled with a de-identified participant code and stored separately from accompanying personal health information. Collection and retention of blood samples will be documented by study staff to account for all blood samples. Samples will be destroyed within 15 years from study initiation. If the PI/QI moves or retires, samples will be either transferred or destroyed.

6.8 MRI Scanning

Magnetic resonance imaging (MRI) scans will be acquired at the CAMH Research Imaging Centre on a GE Discovery MR750 3T scanner (General Electric Medical Systems, Milwaukee, WI) equipped with an 8-channel headcoil. The MRI session will require no more than 30 minutes in the scanner.

6.9 Baseline Symptom and Adverse Events Review

Baseline symptoms and adverse events (AEs) will be assessed using a structured symptom checklist. Participants will be asked to rate the presence and severity of symptoms in a symptom checklist at the Screening (baseline) visit to assess presence of symptoms prior to study procedures occurring, and also at relevant visits after radiotracer administration to assess for emergent AEs. Any emergent AEs will be recorded by study staff and reviewed by the PI/QI. AEs will be followed-up to their resolution before participants are released from the study.

ASSESSMENT OF SAFETY

7.1 Anticipated Risks

[^{18}F]SynVesT-1 is an investigational positron emitting radiopharmaceutical used for research purposes not yet marketed in Canada. The amount of radiotracer injected is very small (microgram) and would not be expected to interfere with the normal functioning of the brain and the body based on experience with a larger number of research on many radiotracers that bind to SV2A, and medications that bind to SV2A. The present study will use 185 MBq for the [^{18}F]SDM8 PET scan (+/-10%), the typical amount applied in neuroimaging studies. There are no known contraindications to this radiotracer, including possible drug interactions, when used in tracer quantity. Presently this radiotracer is being applied in many sites across the world including Yale and Imperial College. It is not known how [^{18}F]SDM8 may affect a fetus or how [^{18}F]SDM8 is excreted in human breast milk. As such, females who are pregnant, would like to become pregnant during the course of the study, or are breastfeeding will be excluded from the study. Although no drug related adverse event has been reported with [^{18}F]SDM8, it is prudent not to administer [^{18}F]SDM8 Inj to individuals who have a history of severe allergic reactions. The formulation contains ethanol and thus might be contraindicated in individuals with an alcohol allergy, an alcohol dehydrogenase 2 deficiency (Asian Flushing Syndrome) or an alcohol use disorder (especially if taking an alcohol or opioid antagonist).

[^{11}C]-(+)-DTBZ is an investigational positron emitting radiopharmaceutical used for research purposes not yet marketed in Canada. [^{11}C]-(+)-DTBZ has been safely used in over 1,000 scans with no pharmacological effect or attributable adverse event. The IB lists no known contraindications. The present study will use 370 MBq for the [^{11}C]-(+)-DTBZ PET scan (+/-10%), within the recommended adult dose listed in the IB as 350 - 700 MBq range. There are no known contraindications to this radiotracer, including possible drug interactions, when used in tracer quantity. It is not known how [^{11}C]-(+)-DTBZ may affect a fetus or how [^{11}C]-(+)-DTBZ is excreted in human breast milk. As such, females who are pregnant, would like to become pregnant during the course of the study, or are breastfeeding will be excluded from the study. Although no drug related adverse event has been reported with [^{11}C]-(+)-DTBZ, it is prudent not to administer [^{11}C]-(+)-DTBZ Inj to individuals who have a history of severe allergic reactions.

7.2 Adverse Events

7.2.1 Methods and timing for assessing, recording, and analyzing safety parameters

Adverse events (AEs) will be recorded in the study Adverse Event Log. The Principal Investigator/Qualified Investigator (PI/QI) will be consulted to make a determination on the reasonable causal relationship to the investigational product and the seriousness of the event.

7.2.2 Procedures for eliciting reports of and for recording and reporting adverse events

Where the event is deemed to meet CAMH REB reporting criteria, the PI/QI will notify the REB. Every adverse event recorded in the participant's file will be assessed by the PI/QI or

another physician involved in the study. The PI/QI will report all Serious and Unexpected Adverse Drug Reactions (SUADR) to Health Canada as per Division 5 regulations.

7.3 Assessment and Follow-up

A follow up phone call will be made 1-2 weeks after each PET scan to check on arterial line recovery and collect data on AEs. Any emergent AEs will be followed to resolution. After study completion (i.e. withdrawal at any time, including voluntary, PI/QI-initiated, or study completers) the participant will be referred back to the care of their regular treating physician, or if the participant does not have a regular treating physician, the PI/QI will provide advice as to how to find one and provide routine psychiatric treatment through this transition.
The study will end after the date of the last visit of the last participant, including the completion of any follow-ups, monitoring and data collection.

8. DATA ANALYSIS

8.1 PET Image Analysis for Main Objectives

Time Activity Curves from Regions of Interest

The primary ROI are ventral striatum and dorsal putamen. For calculating VMAT2 BP_{ND}, occipital cortex is the reference region and the caudate and midbrain ROI will also be measured. VMAT2 levels are very low in other brain regions. In addition to aforementioned regions, SV2A V_T will be measured in hippocampus, ACC, prefrontal cortex (PFC) and subregions including dorsolateral, ventrolateral, orbito- and medial PFC; posterior cingulate, insula, parietal and temporal cortex and thalamus. ROI delineation and analysis uses in-house image analysis software (ROMI)[139]. It applies a standard brain template (Montreal Neurological Institute 152 MRI) containing the predefined ROIs (based on the atlas of Duvernoy[140] but consistent with the atlas of Talairach[141]; striatal divisions from Mawlawi et al.[142] and PFC subregions from cytoarchitectural definitions of Rajkowska[42, 143]). These ROI are transformed and deformed (SPM normalization); to fit each individual T_1 MRI. Each individual's set of ROIs is then iteratively refined based on the probability of each voxel belonging to grey matter (SPM8 segmentation). Each MRI is co-registered to the summed PET image using normalized mutual information algorithm implemented in SPM8 [144]. The transformation is applied to the individual's refined ROIs and resliced to the dimensions of the PET image. Our pipeline includes motion correction and options for partial volume effect correction[145].

Depending on the most optimal quantification for each region, ROI delineation and analysis is performed by using in-house image templates on PMOD software or in-house image analysis software for automated quantification of PET data (ROMI)[80]. It applies a standard brain template (International Consortium for Brain Mapping/Montreal Neurological Institute 152 MRI) containing a set of predefined cortical and subcortical ROIs (based on the atlas of Duvernoy[81] but consistent with the atlas of Talairach[82]; striatal divisions are from Mawlawi et al.[83] and the PFC subregions from the cytoarchitectural definitions of Rajkowska as applied in our previous papers[76, 84]). These ROI are transformed and deformed (SPM normalization and co-registration); to fit each individual T_1 MRI. Each individual's set of ROIs is then iteratively refined based on the probability of each voxel belonging to grey matter (SPM8 segmentation). Each MRI is co-registered to the summed PET image using a normalized mutual information algorithm implemented in SPM8 [85]. The transformation is applied to the individual's refined ROIs and then resliced to match the dimensions of the PET images. Our pipeline includes motion correction and options for partial volume effect correction[86].

Measurement of SV2A V_T and VMAT2 BP_{ND} will prioritize validated modeling methods[55, 135]. SV2A V_T measurement will apply a 1 tissue compartment model with arterial plasma [^{18}F]SynVesT1 radioactivity as the input function to region of interest data[55]. VMAT2 BP_{ND} measurement will use occipital cortex as reference region with the logan method[135] and will also assess using SRTM[146].

8.2 Statistical Analyses

For hypothesis 1 and 2, we will apply linear mixed-effects model (LMM) and treat the regional VMAT2 BP_{ND} (ventral striatum, dorsal putamen) or the regional SV2A V_T (ventral striatum, dorsal putamen) as correlated response variables in their respective models. LMM provides flexibility in both the mean structure and correlation matrix for the targeted outcome measured in multiple brain regions. Group membership (COVID-DNP vs healthy controls) will be the primary predictors. Brain region and group by brain region interaction will be included to account for regional differences. Key demographics (like age) or baseline clinic characteristics may also be controlled in the LMM if necessary and applied to adjust the Pearson correlation coefficient for hypotheses 3 and 4.

For hypothesis 3 and 4, in the COVID-DNP group, the Pearson correlation coefficient for VMAT2 BP_{ND} and SV2A V_T in ventral striatum will be examined in relation to measures of reward listed in section 3.4 (**SHAPS**, ACIPS, EEfRT, delayed discount task, BAS/BIS reward subscale). Similarly, the Pearson correlation coefficient for VMAT2 BP_{ND} and SV2A V_T in dorsal striatum will be examined in relation to measures of motor speed, cognitive processing speed and motivation listed in section 3.4 (**finger tapping test**, comprehensive trail making test, digit symbol substitution, lassitude scale of IDAS, BAS drive subscale). While all measures are of interest, the prioritized measures are bolded above. Holm-Bonferroni multiple comparison correction will be applied for secondary measures.

All outcome measures will be inspected first to provide guidance for subsequent analyses in terms of distribution form and missing data pattern. Based on our prior studies we expect a maximum dropout rate of 10% or less. Full information maximum likelihood method will be used to deal with missing data. Diagnostic analysis will also be conducted to assess impact of potential violations of statistical model assumptions including missing-at-random assumption. Spearman's correlation and linear rank regression will be used instead of Pearson correlation if the outcome variable is not normally distributed. All statistical analyses will be done with standard statistical software packages SAS or R.

For exploratory hypothesis (i): Pearson correlation coefficient between VMAT2 BP_{ND} and SV2A V_T in dorsal striatum and ventral striatum will be examined in COVID-DNP. For exploratory hypotheses (ii) and (iii) a similar approach as for hypothesis 1 and 2 will be applied using LMM treating the regional VMAT2 BP_{ND} or the regional SV2A V_T as correlated response variables in their respective models with group membership (COVID-DNP vs healthy controls) as the primary predictor.

Sex based Analyses: For each aim, we will supplement the main analysis with stratified analysis for females alone and males alone. For hypotheses 1 and 2, we will also assess for between sex difference by adding group (COVID-DNP vs healthy controls) by sex interactions for each analysis to evaluate how sex may moderate the association between group and outcomes of interest.

8.3 Power

To assess the power of our study, we have conservatively grouped Aims 1 and 2 due to the similar nature of the hypotheses and assigned alpha = .025 to each aim. For proposed sample size of 50 in each group, we have sufficient power (>0.80) to detect a moderate to large between group difference, Cohen's f of 0.31, of VMAT2 BP_{ND} or SV2A V_T in the two regions of interest (dorsal striatum and ventral striatum). We assumed a correlation of .6 between repeated measures (the ROI in the same participants) and a 10% attrition (i.e. effective sample size of 45 in each group). For sex-based analyses, the minimum detectable group effect increases to 0.45 (Cohen's f) for sex stratified analysis and 0.66 for between sex differences. For the same reason, we have also grouped Aims 3 and 4 and assigned alpha = .025 to each aim. For these two aims, for an effect size ρ=0.45, power is >0.8 to detect correlations and for an effect size ρ=0.55 power is >0.8 to detect correlations within subgroups of different sex. We also accounted for the 10% attrition in the calculation.

9. DATA MANAGEMENT AND RETENTION

The case report forms (CRFs) used to capture the data is REDCap. The study data recorded on REDCap will be derived from source documents (SDs) and will be consistent with the SDs. Any discrepancies will be explained, and any changes or corrections will be documented in writing and approved by the study QI.

All data collected about the participants in the context of the study will be kept strictly confidential and will remain coded. All information regarding personal health information will be kept strictly confidential and separate from the study data. Data acquired from this study may be combined with data acquired from other studies in Dr. Meyer's lab. Any reports or publications that result from this study will not contain information that will permit identification of participants. De-identified study data and aggregate results may be combined with study databases, either from projects overseen by the PI/QI or externally held databases, with the express approval of CAMH REB. The informed consent form will clearly outline details on data sharing and future use of samples using de-identified study data.

Data and all appropriate research documentation will be stored for a minimum of 15 years after the completion of the study, including the follow-up period.

Study data collected prior to participant withdrawal will be retained unless the participant requests complete withdrawal of study data. The participant may also request that their blood samples be destroyed at any time. The PI/QI will ensure that destruction of biological samples be completed within a reasonable timeframe and will no longer be used for research immediately upon withdrawal of consent. Participant study data cannot be withdrawn once it has been published.

10. REGULATORY ASPECTS

10.1 Ethics Approval

The PI/QI will obtain approvals from the CAMH REB, and Health Canada prior to the initiation of any study procedures.

10.2 Consent

Consent to enter the study must be sought from each participant only after a full explanation has been given, a consent form offered, and time allowed for consideration. Signed participant consent will be obtained, as this is mandatory for this study. The right of the participant to refuse to participate without giving reasons must be respected. All participants are free to withdraw at any time from the protocol treatment without giving reasons and without prejudicing further treatment.

10.3 Confidentiality

The Investigator will preserve the confidentiality of all study participant records and regulatory files are kept confidential and are stored in a secure, limited-access location in accordance with CAMH policies, PHIPA, Tri-Council Policy Statement (TCPS2) and the International Conference on Harmonization Guideline for Good Clinical Practice (ICH GCP) requirements. The study staff will protect participants' confidentiality to the extent permitted by law.

A description of this research study might be published in public registries of clinical trials such as ClinicalTrials.gov (http://www.clinicaltrials.gov). No information that identifies study participants will be sent to these registries. At most, these registries will publish a summary of the study results. Participants' de-identified study data and results could be sent to research collaborators working with us at sites around the world; if this occurs, the participant's research identification will be used and identifiable data (e.g., name and date of birth) will not be passed on.

10.4 Sponsor

This is an independent, investigator-initiated study. The Brain Health Imaging Centre is the sponsor.

10.5 Funding

Funding is from CIHR.

10.6 Monitoring

This study will have trial-related monitoring that will be conducted according to the study-monitoring plan. The study will be monitored according to CAMH policy.

10.7 Audits

The study may be subject to inspection and audit by CAMH Quality Assurance, CAMH REB and Health Canada.

REFERENCES

1. Lu R, Zhao X, Li J, Niu P, Yang B, Wu H, Wang W, Song H, Huang B, Zhu N, Bi Y, Ma X, Zhan F, Wang L, Hu T, Zhou H, Hu Z, Zhou W, Zhao L, Chen J, Meng Y, Wang J, Lin Y, Yuan J, Xie Z, Ma J, Liu WJ, Wang D, Xu W, Holmes EC, Gao GF, Wu G, Chen W, Shi W, Tan W. Genomic characterisation and epidemiology of 2019 novel coronavirus: implications for virus origins and receptor binding. *Lancet.* 2020;395(10224):565-574.

2. Houhamdi L, Gautret P, Hoang VT, Fournier PE, Colson P, Raoult D. Characteristics of the first 1119 SARS-CoV-2 Omicron variant cases, in Marseille, France, November-December 2021. *J Med Virol.* 2022;94(5):2290-2295.

3. Ulloa AC, Buchan SA, Daneman N, Brown KA. Estimates of SARS-CoV-2 Omicron Variant Severity in Ontario, Canada. *JAMA.* 2022;327(13):1286-1288.

4. Wolter N, Jassat W, Walaza S, Welch R, Moultrie H, Groome M, Amoako DG, Everatt J, Bhiman JN, Scheepers C, Tebeila N, Chiwandire N, du Plessis M, Govender N, Ismail A, Glass A, Mlisana K, Stevens W, Treurnicht FK, Makatini Z, Hsiao NY, Parboosing R, Wadula J, Hussey H, Davies MA, Boulle A, von Gottberg A, Cohen C. Early assessment of the clinical severity of the SARS-CoV-2 omicron variant in South Africa: a data linkage study. *Lancet.* 2022;399(10323):437-446.

5. Centers_for_Disease_Control_and_Prevention. Omicron Variant: What you need to know. https://www.cdc.gov/coronavirus/2019-ncov/variants/omicron-variant.html. Accessed July 29, 2022.

6. WHO. Epidemiology and Clinical Management of COVID-19. CORONAVIRUS (COVID-19) UPDATE NO. 31 19 June 2020

https://www.who.int/docs/default-source/coronaviruse/risk-comms-updates/update-31-epidemiology-clinical-mgt.pdf?sfvrsn=2cecb43c_2.

7. Wu Z, McGoogan JM. Characteristics of and Important Lessons From the Coronavirus Disease 2019 (COVID-19) Outbreak in China: Summary of a Report of 72314 Cases From the Chinese Center for Disease Control and Prevention. *JAMA.* 2020;323(13):1239-1242.

8. Centre_for_Disease_Control. COVID-19. https://www.cdc.gov/coronavirus/2019-ncov/covid-data/covidview/index.html. Accessed May 12, 2022.

9. WHO. World Health Organization Coronavirus (COVID-19) Dashboard. September 14, 2022; https://covid19.who.int/?mapFilter=cases. Accessed September 15, 2022, 2022.

10. Varatharaj A, Thomas N, Ellul MA, Davies NWS, Pollak TA, Tenorio EL, Sultan M, Easton A, Breen G, Zandi M, Coles JP, Manji H, Al-Shahi Salman R, Menon DK, Nicholson TR, Benjamin LA, Carson A, Smith C, Turner MR, Solomon T, Kneen R, Pett SL, Galea I, Thomas RH, Michael BD, CoroNerve Study G. Neurological and neuropsychiatric complications of COVID-19 in 153 patients: a UK-wide surveillance study. *Lancet Psychiatry.* 2020.

11. Ellul MA, Benjamin L, Singh B, Lant S, Michael BD, Easton A, Kneen R, Defres S, Sejvar J, Solomon T. Neurological associations of COVID-19. *Lancet Neurol.* 2020.

12. Rogers JP, Chesney E, Oliver D, Pollak TA, McGuire P, Fusar-Poli P, Zandi MS, Lewis G, David AS. Psychiatric and neuropsychiatric presentations associated with severe

coronavirus infections: a systematic review and meta-analysis with comparison to the COVID-19 pandemic. *Lancet Psychiatry.* 2020;7(7):611-627.
13. Office_of_National_Statistics_(United_Kingdom). Self-reported long COVID after infection with the Omicron variant in the UK: 6 May 2022. 2022.
14. Menni C, Klaser K, May A, Polidori L, Capdevila J, Louca P, Sudre CH, Nguyen LH, Drew DA, Merino J, Hu C, Selvachandran S, Antonelli M, Murray B, Canas LS, Molteni E, Graham MS, Modat M, Joshi AD, Mangino M, Hammers A, Goodman AL, Chan AT, Wolf J, Steves CJ, Valdes AM, Ourselin S, Spector TD. Vaccine side-effects and SARS-CoV-2 infection after vaccination in users of the COVID Symptom Study app in the UK: a prospective observational study. *Lancet Infect Dis.* 2021;21(7):939-949.
15. Kuodi P, Gorelik Y, Zayyad H, Wertheim O, Wiegler K, Jabal K, Dror A, Nazzai S, Glikman D, Edlelstein M. Association between vaccination status and reported incidence of post-acute COVID-19 symptoms in Isreal: a cross-sectional study of patients tested between March 2020 and November 2021. *medRxiv preprint.* 2021.
16. Al-Aly Z, Bowe B, Xie Y. Long COVID after breakthrough SARS-CoV-2 infection. *Nat Med.* 2022.
17. Antonelli M, Pujol JC, Spector TD, Ourselin S, Steves CJ. Risk of long COVID associated with delta versus omicron variants of SARS-CoV-2. *Lancet.* 2022;399(10343):2263-2264.
18. Centers_for_Disease_Control_and_Prevention. Long COVID or Post-Covid Conditions. https://www.cdc.gov/coronavirus/2019-ncov/long-term-effects/.
19. Al-Aly Z, Bowe B, Xie Y. Outcomes of SARS-CoV-2 Reinfection. *Research Square (Preprint).* 2022.
20. Davis HE, Assaf GS, McCorkell L, Wei H, Low RJ, Re'em Y, Redfield S, Austin JP, Akrami A. Characterizing long COVID in an international cohort: 7 months of symptoms and their impact. *EClinicalMedicine.* 2021;38:101019.
21. Taquet M, Sillett R, Zhu L, Mendel J, Camplisson I, Dercon Q, Harrison PJ. Neurological and psychiatric risk trajectories after SARS-CoV-2 infection: an analysis of 2-year retrospective cohort studies including 1 284 437 patients. *Lancet Psychiatry.* 2022.
22. Lamontagne SJ, Winters MF, Pizzagalli DA, Olmstead MC. Post-acute sequelae of COVID-19: Evidence of mood & cognitive impairment. *Brain Behav Immun Health.* 2021;17:100347.
23. Subramanian A, Nirantharakumar K, Hughes S, Myles P, Williams T, Gokhale KM, Taverner T, Chandan JS, Brown K, Simms-Williams N, Shah AD, Singh M, Kidy F, Okoth K, Hotham R, Bashir N, Cockburn N, Lee SI, Turner GM, Gkoutos GV, Aiyegbusi OL, McMullan C, Denniston AK, Sapey E, Lord JM, Wraith DC, Leggett E, Iles C, Marshall T, Price MJ, Marwaha S, Davies EH, Jackson LJ, Matthews KL, Camaradou J, Calvert M, Haroon S. Symptoms and risk factors for long COVID in non-hospitalized adults. *Nat Med.* 2022;28(8):1706-1714.
24. Lopez-Leon S, Wegman-Ostrosky T, Perelman C, Sepulveda R, Rebolledo PA, Cuapio A, Villapol S. More than 50 long-term effects of COVID-19: a systematic review and meta-analysis. *Sci Rep.* 2021;11(1):16144.
25. Da Costa B, Yasuda C, Cendes F, Aventurato I. Worsening of neuropsychiatric symptoms after six months from the acute COVID-19 infection in 1183 subjects. *Journal of the Neurological Sciences.* 2021;429:129.

26. Blackmon K, Day GS, Powers HR, Bosch W, Prabhakaran D, Woolston D, Pedraza O. Neurocognitive screening in patients following SARS-CoV-2 infection: tools for triage. *BMC Neurol.* 2022;22(1):285.
27. Government_of_Canada. Coronavirus Disease (COVID-19): Epidemiology Update. https://health-infobase.canada.ca/covid-19/epidemiological-summary-covid-19-cases.html. Accessed May 12, 2022.
28. Government_of_Canada. Government of Canada/Coronavirus Disease (COVID-19): Outbreak update. https://www.canada.ca/en/public-health/services/diseases/2019-novel-coronavirus-infection.html.
29. DosSantos MF, Devalle S, Aran V, Capra D, Roque NR, Coelho-Aguiar JM, Spohr T, Subilhaga JG, Pereira CM, D'Andrea Meira I, Niemeyer Soares Filho P, Moura-Neto V. Neuromechanisms of SARS-CoV-2: A Review. *Front Neuroanat.* 2020;14:37.
30. Boldrini M, Canoll PD, Klein RS. How COVID-19 Affects the Brain. *JAMA Psychiatry.* 2021;78(6):682-683.
31. Torices S, Cabrera R, Stangis M, Naranjo O, Fattakhov N, Teglas T, Adesse D, Toborek M. Expression of SARS-CoV-2-related receptors in cells of the neurovascular unit: implications for HIV-1 infection. *J Neuroinflammation.* 2021;18(1):167.
32. Chen R, Wang K, Yu J, Chen Z, Wen C, Xu Z. The spatial and cell-type distribution of SARS-CoV-2 receptor ACE2 in human and mouse brain. *bioRxiv preprint.* 2020;https://www.biorxiv.org/content/biorxiv/early/2020/04/09/2020.04.07.030650.full.pdf.
33. Dantzer R, O'Connor JC, Freund GG, Johnson RW, Kelley KW. From inflammation to sickness and depression: when the immune system subjugates the brain. *Nat Rev Neurosci.* 2008;9(1):46-56.
34. Coolen T, Lolli V, Sadeghi N, Rovai A, Trotta N, Taccone F, Creteur J, Henrard S, Goffard J-C, De Witte O, Naeije G, Goldman S, De Tiege X. Early Postmortem Brain MRI Findings in COVID-19 non-survivors. *Neurology.* in press.
35. Tremblay ME, Madore C, Bordeleau M, Tian L, Verkhratsky A. Neuropathobiology of COVID-19: The Role for Glia. *Front Cell Neurosci.* 2020;14:592214.
36. Matschke J, Lutgehetmann M, Hagel C, Sperhake JP, Schroder AS, Edler C, Mushumba H, Fitzek A, Allweiss L, Dandri M, Dottermusch M, Heinemann A, Pfefferle S, Schwabenland M, Sumner Magruder D, Bonn S, Prinz M, Gerloff C, Puschel K, Krasemann S, Aepfelbacher M, Glatzel M. Neuropathology of patients with COVID-19 in Germany: a post-mortem case series. *Lancet Neurol.* 2020;19(11):919-929.
37. Cosentino G, Todisco M, Hota N, Della Porta G, Morbini P, Tassorelli C, Pisani A. Neuropathological findings from COVID-19 patients with neurological symptoms argue against a direct brain invasion of SARS-CoV-2: A critical systematic review. *Eur J Neurol.* 2021;28(11):3856-3865.
38. Rutkai I, Mayer MG, Hellmers LM, Ning B, Huang Z, Monjure CJ, Coyne C, Silvestri R, Golden N, Hensley K, Chandler K, Lehmicke G, Bix GJ, Maness NJ, Russell-Lodrigue K, Hu TY, Roy CJ, Blair RV, Bohm R, Doyle-Meyers LA, Rappaport J, Fischer T. Neuropathology and virus in brain of SARS-CoV-2 infected non-human primates. *Nat Commun.* 2022;13(1):1745.
39. Meyer J. Applying PET Markers of Gliosis for Disease Stratification, Target Engagement and Characterization of State. Paper presented at: American College of Neuropsychopharmacology, 2020; Virtual Meeting.

40. Moriguchi S, Wilson AA, Miler L, Rusjan PM, Vasdev N, Kish SJ, Rajkowska G, Wang J, Bagby M, Mizrahi R, Varughese B, Houle S, Meyer JH. Monoamine Oxidase B Total Distribution Volume in the Prefrontal Cortex of Major Depressive Disorder: An [11C]SL25.1188 Positron Emission Tomography Study. *JAMA Psychiatry.* 2019.

41. Attwells S, Setiawan E, Wilson AA, Rusjan PM, Mizrahi R, Miler L, Xu C, Richter MA, Kahn A, Kish SJ, Houle S, Ravindran L, Meyer JH. Inflammation in the Neurocircuitry of Obsessive-Compulsive Disorder. *JAMA Psychiatry.* 2017;74(8):833-840.

42. Setiawan E, Wilson AA, Mizrahi R, Rusjan PM, Miler L, Rajkowska G, Suridjan I, Kennedy JL, Rekkas PV, Houle S, Meyer JH. Role of translocator protein density, a marker of neuroinflammation, in the brain during major depressive episodes. *JAMA Psychiatry.* 2015;72(3):268-275.

43. Meyer JH, Ginovart N, Boovariwala A, Sagrati S, Hussey D, Garcia A, Young T, Praschak-Rieder N, Wilson AA, Houle S. Elevated monoamine oxidase a levels in the brain: an explanation for the monoamine imbalance of major depression. *Arch Gen Psychiatry.* 2006;63(11):1209-1216.

44. Chen Z, Jalabi W, Hu W, Park HJ, Gale JT, Kidd GJ, Bernatowicz R, Gossman ZC, Chen JT, Dutta R, Trapp BD. Microglial displacement of inhibitory synapses provides neuroprotection in the adult brain. *Nat Commun.* 2014;5:4486.

45. Kettenmann H, Kirchhoff F, Verkhratsky A. Microglia: new roles for the synaptic stripper. *Neuron.* 2013;77(1):10-18.

46. Tremblay ME. The role of microglia at synapses in the healthy CNS: novel insights from recent imaging studies. *Neuron Glia Biol.* 2011;7(1):67-76.

47. Rajendran L, Paolicelli RC. Microglia-Mediated Synapse Loss in Alzheimer's Disease. *J Neurosci.* 2018;38(12):2911-2919.

48. Lee E, Chung WS. Glial Control of Synapse Number in Healthy and Diseased Brain. *Front Cell Neurosci.* 2019;13:42.

49. Rial D, Lemos C, Pinheiro H, Duarte JM, Goncalves FQ, Real JI, Prediger RD, Goncalves N, Gomes CA, Canas PM, Agostinho P, Cunha RA. Depression as a Glial-Based Synaptic Dysfunction. *Front Cell Neurosci.* 2015;9:521.

50. Kang HJ, Voleti B, Hajszan T, Rajkowska G, Stockmeier CA, Licznerski P, Lepack A, Majik MS, Jeong LS, Banasr M, Son H, Duman RS. Decreased expression of synapse-related genes and loss of synapses in major depressive disorder. *Nat Med.* 2012;18(9):1413-1417.

51. Duric V, Banasr M, Stockmeier CA, Simen AA, Newton SS, Overholser JC, Jurjus GJ, Dieter L, Duman RS. Altered expression of synapse and glutamate related genes in post-mortem hippocampus of depressed subjects. *Int J Neuropsychopharmacol.* 2013;16(1):69-82.

52. Lynch BA, Lambeng N, Nocka K, Kensel-Hammes P, Bajjalieh SM, Matagne A, Fuks B. The synaptic vesicle protein SV2A is the binding site for the antiepileptic drug levetiracetam. *Proc Natl Acad Sci U S A.* 2004;101(26):9861-9866.

53. Bragina L, Fattorini G, Giovedi S, Melone M, Bosco F, Benfenati F, Conti F. Analysis of Synaptotagmin, SV2, and Rab3 Expression in Cortical Glutamatergic and GABAergic Axon Terminals. *Front Cell Neurosci.* 2011;5:32.

54. Finnema SJ, Nabulsi NB, Eid T, Detyniecki K, Lin SF, Chen MK, Dhaher R, Matuskey D, Baum E, Holden D, Spencer DD, Mercier J, Hannestad J, Huang Y, Carson RE.

Imaging synaptic density in the living human brain. *Sci Transl Med.* 2016;8(348):348ra396.

55. Naganawa M, Li S, Nabulsi N, Henry S, Zheng MQ, Pracitto R, Cai Z, Gao H, Kapinos M, Labaree D, Matuskey D, Huang Y, Carson RE. First-in-Human Evaluation of (18)F-SynVesT-1, a Radioligand for PET Imaging of Synaptic Vesicle Glycoprotein 2A. *J Nucl Med.* 2021;62(4):561-567.

56. Li S, Naganawa M, Pracitto R, Najafzadeh S, Holden D, Henry S, Matuskey D, Emery PR, Cai Z, Ropchan J, Nabulsi N, Carson RE, Huang Y. Assessment of test-retest reproducibility of [(18)F]SynVesT-1, a novel radiotracer for PET imaging of synaptic vesicle glycoprotein 2A. *Eur J Nucl Med Mol Imaging.* 2021;48(5):1327-1338.

57. Zhao N, Li W, Zhang SF, Yang BX, Sha S, Cheung T, Jackson T, Zang YF, Xiang YT. Network Analysis of Depressive Symptoms Among Residents of Wuhan in the Later Stage of the COVID-19 Pandemic. *Front Psychiatry.* 2021;12:735973.

58. Hunter RL, Cheng B, Choi DY, Liu M, Liu S, Cass WA, Bing G. Intrastriatal lipopolysaccharide injection induces parkinsonism in C57/B6 mice. *J Neurosci Res.* 2009;87(8):1913-1921.

59. Choi DY, Liu M, Hunter RL, Cass WA, Pandya JD, Sullivan PG, Shin EJ, Kim HC, Gash DM, Bing G. Striatal neuroinflammation promotes Parkinsonism in rats. *PLoS One.* 2009;4(5):e5482.

60. Deng I, Corrigan F, Garg S, Zhou XF, Bobrovskaya L. Further Characterization of Intrastriatal Lipopolysaccharide Model of Parkinson's Disease in C57BL/6 Mice. *Int J Mol Sci.* 2021;22(14).

61. Kish SJ, Robitaille Y, el-Awar M, Clark B, Schut L, Ball MJ, Young LT, Currier R, Shannak K. Striatal monoamine neurotransmitters and metabolites in dominantly inherited olivopontocerebellar atrophy. *Neurology.* 1992;42(8):1573-1577.

62. Koeppe RA, Frey KA, Vander Borght TM, Karlamangla A, Jewett DM, Lee LC, Kilbourn MR, Kuhl DE. Kinetic evaluation of [11C]dihydrotetrabenazine by dynamic PET: measurement of vesicular monoamine transporter. *J Cereb Blood Flow Metab.* 1996;16(6):1288-1299.

63. Kilbourn MR, Koeppe RA. A six-year longitudinal PET study of (+)-[(11)C]DTBZ binding to the VMAT2 in monkey brain. *Nucl Med Biol.* 2017;55:34-37.

64. Kilbourn MR, Kuszpit K, Sherman P. Rapid and differential losses of in vivo dopamine transporter (DAT) and vesicular monoamine transporter (VMAT2) radioligand binding in MPTP-treated mice. *Synapse.* 2000;35(4):250-255.

65. Miller GW, Erickson JD, Perez JT, Penland SN, Mash DC, Rye DB, Levey AI. Immunochemical analysis of vesicular monoamine transporter (VMAT2) protein in Parkinson's disease. *Exp Neurol.* 1999;156(1):138-148.

66. Fu JF, Klyuzhin I, McKenzie J, Neilson N, Shahinfard E, Dinelle K, McKeown MJ, Stoessl AJ, Sossi V. Joint pattern analysis applied to PET DAT and VMAT2 imaging reveals new insights into Parkinson's disease induced presynaptic alterations. *Neuroimage Clin.* 2019;23:101856.

67. Frey KA, Koeppe RA, Kilbourn MR, Vander Borght TM, Albin RL, Gilman S, Kuhl DE. Presynaptic monoaminergic vesicles in Parkinson's disease and normal aging. *Ann Neurol.* 1996;40(6):873-884.

68. Gilman S, Frey KA, Koeppe RA, Junck L, Little R, Vander Borght TM, Lohman M, Martorello S, Lee LC, Jewett DM, Kilbourn MR. Decreased striatal monoaminergic

terminals in olivopontocerebellar atrophy and multiple system atrophy demonstrated with positron emission tomography. *Ann Neurol.* 1996;40(6):885-892.

69. Suzuki M, Desmond TJ, Albin RL, Frey KA. Cholinergic vesicular transporters in progressive supranuclear palsy. *Neurology.* 2002;58(7):1013-1018.

70. Meyer JH, Kruger S, Wilson AA, Christensen BK, Goulding VS, Schaffer A, Minifie C, Houle S, Hussey D, Kennedy SH. Lower dopamine transporter binding potential in striatum during depression. *Neuroreport.* 2001;12(18):4121-4125.

71. Meyer JH, McNeely HE, Sagrati S, Boovariwala A, Martin K, Verhoeff NP, Wilson AA, Houle S. Elevated putamen D(2) receptor binding potential in major depression with motor retardation: an [11C]raclopride positron emission tomography study. *Am J Psychiatry.* 2006;163(9):1594-1602.

72. Tavares-Junior JWL, de Souza ACC, Borges JWP, Oliveira DN, Siqueira-Neto JI, Sobreira-Neto MA, Braga-Neto P. COVID-19 associated cognitive impairment: A systematic review. *Cortex.* 2022;152:77-97.

73. National_Health_Service(UK). Long COVID: Fatigue. https://www.nhsinform.scot/long-term-effects-of-covid-19-long-covid/signs-and-symptoms/long-covid-fatigue. Accessed August 1, 2022.

74. Schultz W. Reward functions of the basal ganglia. *J Neural Transm (Vienna).* 2016;123(7):679-693.

75. Daniel R, Pollmann S. A universal role of the ventral striatum in reward-based learning: evidence from human studies. *Neurobiol Learn Mem.* 2014;114:90-100.

76. Haber S. Neuroanatomy of Reward: A View from the Ventral Striatum. In: Gottfried J, ed. *Neurobiology of Sensation and Reward*. Boca Raton (FL): CRC Press/Taylor and Francis; 2011.

77. Ghandili M, Munakomi S. Neuroanatomy, Putamen. *StatPearls*. Treasure Island (FL); 2022.

78. Liljeholm M, O'Doherty JP. Contributions of the striatum to learning, motivation, and performance: an associative account. *Trends Cogn Sci.* 2012;16(9):467-475.

79. McGuigan S, Zhou SH, Brosnan MB, Thyagarajan D, Bellgrove MA, Chong TT. Dopamine restores cognitive motivation in Parkinson's disease. *Brain.* 2019;142(3):719-732.

80. Armstrong MJ, Okun MS. Diagnosis and Treatment of Parkinson Disease: A Review. *JAMA.* 2020;323(6):548-560.

81. Loas G, Krystkowiak P, Godefroy O. Anhedonia in Parkinson's disease: an overview. *J Neuropsychiatry Clin Neurosci.* 2012;24(4):444-451.

82. Alberico SL, Cassell MD, Narayanan NS. The Vulnerable Ventral Tegmental Area in Parkinson's Disease. *Basal Ganglia.* 2015;5(2-3):51-55.

83. Snow BJ, Vingerhoets FJ, Langston JW, Tetrud JW, Sossi V, Calne DB. Pattern of dopaminergic loss in the striatum of humans with MPTP induced parkinsonism. *J Neurol Neurosurg Psychiatry.* 2000;68(3):313-316.

84. Langston JW, Ballard P, Tetrud JW, Irwin I. Chronic Parkinsonism in humans due to a product of meperidine-analog synthesis. *Science.* 1983;219(4587):979-980.

85. Brown CA, Campbell MC, Karimi M, Tabbal SD, Loftin SK, Tian LL, Moerlein SM, Perlmutter JS. Dopamine pathway loss in nucleus accumbens and ventral tegmental area predicts apathetic behavior in MPTP-lesioned monkeys. *Exp Neurol.* 2012;236(1):190-197.

86. Fabbrini G, Fabbrini A, Suppa A. Progressive supranuclear palsy, multiple system atrophy and corticobasal degeneration. In: Reus V, Lindqvist D, eds. *Handbook of Clinical Neurology.* Vol 165: Elsevier; 2019:155-177.
87. Kaasinen V, Kankare T, Joutsa J, Vahlberg T. Presynaptic Striatal Dopaminergic Function in Atypical Parkinsonism: A Metaanalysis of Imaging Studies. *J Nucl Med.* 2019;60(12):1757-1763.
88. Gilman S. Biochemical changes in multiple system atrophy detected with positron emission tomography. *Parkinsonism Relat Disord.* 2001;7(3):253-256.
89. Chen R, Wang K, Yu J, Howard D, French L, Chen Z, Wen C, Xu Z. The Spatial and Cell-Type Distribution of SARS-CoV-2 Receptor ACE2 in the Human and Mouse Brains. *Front Neurol.* 2020;11:573095.
90. Hernandez VS, Zetter MA, Guerra EC, Hernandez-Araiza I, Karuzin N, Hernandez-Perez OR, Eiden LE, Zhang L. ACE2 expression in rat brain: Implications for COVID-19 associated neurological manifestations. *Exp Neurol.* 2021;345:113837.
91. Kilbourn M. PET Imaging of Vesicular Monoamine Transporters. In: Dierckx R, Otte A, de Vries E, van Waarde A, eds. *PET and SPECT of Neurobiological Systems*. Second Edition ed. Switzerland: Springer; 2020:1091-1131.
92. Van Camp N, Lavisse S, Roost P, Gubinelli F, Hillmer A, Boutin H. TSPO imaging in animal models of brain diseases. *Eur J Nucl Med Mol Imaging.* 2021;49(1):77-109.
93. Raval NR, Gudmundsen F, Juhl M, Andersen IV, Speth N, Videbaek A, Petersen IN, Mikkelsen JD, Fisher PM, Herth MM, Plaven-Sigray P, Knudsen GM, Palner M. Synaptic Density and Neuronal Metabolic Function Measured by Positron Emission Tomography in the Unilateral 6-OHDA Rat Model of Parkinson's Disease. *Front Synaptic Neurosci.* 2021;13:715811.
94. Rusjan PM, Wilson AA, Miler L, Fan I, Mizrahi R, Houle S, Vasdev N, Meyer JH. Kinetic modeling of the monoamine oxidase B radioligand [^{11}C]SL25.1188 in human brain with high-resolution positron emission tomography. *J Cereb Blood Flow Metab.* 2014;34(5):883-889.
95. Freedman NM, Mishani E, Krausz Y, Weininger J, Lester H, Blaugrund E, Ehrlich D, Chisin R. In vivo measurement of brain monoamine oxidase B occupancy by rasagiline, using (11)C-l-deprenyl and PET. *J Nucl Med.* 2005;46(10):1618-1624.
96. Chang HY, Li YY, Hong CT, Kuan YC. Efficacy of rasagiline monotherapy for early Parkinson disease: A systematic review and meta-analysis of randomized controlled trials. *J Psychopharmacol.* 2022;36(6):704-714.
97. Youdim MB. Rasagiline in Parkinson's disease. *N Engl J Med.* 2010;362(7):657-658; author reply 658-659.
98. Robinson DS, Amsterdam JD. The selegiline transdermal system in major depressive disorder: a systematic review of safety and tolerability. *J Affect Disord.* 2008;105(1-3):15-23.
99. Haefely W, Burkard WP, Cesura AM, Kettler R, Lorez HP, Martin JR, Richards JG, Scherschlicht R, Da Prada M. Biochemistry and pharmacology of moclobemide, a prototype RIMA. *Psychopharmacology (Berl).* 1992;106 Suppl:S6-14.
100. Chiuccariello L, Cooke RG, Miler L, Levitan RD, Baker GB, Kish SJ, Kolla NJ, Rusjan PM, Houle S, Wilson AA, Meyer JH. Monoamine Oxidase-A Occupancy by Moclobemide and Phenelzine: Implications for the Development of Monoamine Oxidase Inhibitors. *Int J Neuropsychopharmacol.* 2015;19(1).

101. Meyer JH. Applying neuroimaging ligands to study major depressive disorder. *Semin Nucl Med.* 2008;38(4):287-304.
102. Meyer JH, Goulding VS, Wilson AA, Hussey D, Christensen BK, Houle S. Bupropion occupancy of the dopamine transporter is low during clinical treatment. *Psychopharmacology (Berl).* 2002;163(1):102-105.
103. Volkow ND, Wang GJ, Fowler JS, Gatley SJ, Logan J, Ding YS, Hitzemann R, Pappas N. Dopamine transporter occupancies in the human brain induced by therapeutic doses of oral methylphenidate. *Am J Psychiatry.* 1998;155(10):1325-1331.
104. Meyer JH, Wilson AA, Sagrati S, Hussey D, Carella A, Potter WZ, Ginovart N, Spencer EP, Cheok A, Houle S. Serotonin transporter occupancy of five selective serotonin reuptake inhibitors at different doses: an [11C]DASB positron emission tomography study. *Am J Psychiatry.* 2004;161(5):826-835.
105. McIntyre RS, Rosenblat JD, Nemeroff CB, Sanacora G, Murrough JW, Berk M, Brietzke E, Dodd S, Gorwood P, Ho R, Iosifescu DV, Lopez Jaramillo C, Kasper S, Kratiuk K, Lee JG, Lee Y, Lui LMW, Mansur RB, Papakostas GI, Subramaniapillai M, Thase M, Vieta E, Young AH, Zarate CA, Jr., Stahl S. Synthesizing the Evidence for Ketamine and Esketamine in Treatment-Resistant Depression: An International Expert Opinion on the Available Evidence and Implementation. *Am J Psychiatry.* 2021;178(5):383-399.
106. Bahji A, Vazquez GH, Zarate CA, Jr. Comparative efficacy of racemic ketamine and esketamine for depression: A systematic review and meta-analysis. *J Affect Disord.* 2021;278:542-555.
107. Zanos P, Moaddel R, Morris PJ, Georgiou P, Fischell J, Elmer GI, Alkondon M, Yuan P, Pribut HJ, Singh NS, Dossou KS, Fang Y, Huang XP, Mayo CL, Wainer IW, Albuquerque EX, Thompson SM, Thomas CJ, Zarate CA, Jr., Gould TD. NMDAR inhibition-independent antidepressant actions of ketamine metabolites. *Nature.* 2016;533(7604):481-486.
108. Suzuki A, Kunugi A, Tajima Y, Suzuki N, Suzuki M, Toyofuku M, Kuno H, Sogabe S, Kosugi Y, Awasaki Y, Kaku T, Kimura H. Strictly regulated agonist-dependent activation of AMPA-R is the key characteristic of TAK-653 for robust synaptic responses and cognitive improvement. *Sci Rep.* 2021;11(1):14532.
109. Hara H, Suzuki A, Kunugi A, Tajima Y, Yamada R, Kimura H. TAK-653, an AMPA receptor potentiator with minimal agonistic activity, produces an antidepressant-like effect with a favorable safety profile in rats. *Pharmacol Biochem Behav.* 2021;211:173289.
110. ClinicalTrials.gov. Efficacy and Safety of TAK-653 in Treatment-Resistant Depression. https://clinicaltrials.gov/ct2/show/NCT03312894.
111. Hasegawa Y, Zhu X, Kamiya A. NV-5138 as a fast-acting antidepressant via direct activation of mTORC1 signaling. *J Clin Invest.* 2019;129(6):2207-2209.
112. ClinicalTrials.gov. Phase 2 Study of NV-5138 in Adults With Treatment Resistant Depression. https://clinicaltrials.gov/ct2/show/NCT05066672.
113. Holmes SE, Finnema SJ, Naganawa M, DellaGioia N, Holden D, Fowles K, Davis M, Ropchan J, Emory P, Ye Y, Nabulsi N, Matuskey D, Angarita GA, Pietrzak RH, Duman RS, Sanacora G, Krystal JH, Carson RE, Esterlis I. Imaging the effect of ketamine on synaptic density (SV2A) in the living brain. *Mol Psychiatry.* 2022;27(4):2273-2281.

114. First M, Williams J, Karg R, Spitzer R. Structured Clinical Interview for DSM-5-Research Version (SCID-5 for DSM-5, Research Version; SCID-5-RV). In: Association AP, ed. Arlington, VA; 2015.
115. First M, Spitzer R, Williams J, Gibbon M. *"Structured Clinical Interview for DSM-IV Axis I Disorders, Patient Edition (SCID-P), version 2"*. New York: Biometrics Research; 1995.
116. Public_Health_Ontario. Coronavirus Disease 2019 (COVID-19) - PCR. https://www.publichealthontario.ca/en/laboratory-services/test-information-index/covid-19. Accessed September 23, 2020.
117. Government_of_Canada. Clinical Management of Patients with COVID-19: Second interim guidance. https://www.canada.ca/en/public-health/services/diseases/2019-novel-coronavirus-infection/clinical-management-covid-19.html. Accessed October 6, 2020, 2020.
118. World_Health_Organization. Clinical Management of COVID-19: Interim Guidance 27 May 2020; 2020.
119. First M, Williams J, Karg R, Spitzer R. User's Guide for the SCID-5-PD (Structured Clinical Interview for DSM-5 Personality Disorder). In: Association AP, ed. Arlington, VA; 2015.
120. Bernstein DP, Stein JA, Newcomb MD, Walker E, Pogge D, Ahluvalia T, Stokes J, Handelsman L, Medrano M, Desmond D, Zule W. Development and validation of a brief screening version of the Childhood Trauma Questionnaire. *Child Abuse Negl.* 2003;27(2):169-190.
121. Snaith RP, Hamilton M, Morley S, Humayan A, Hargreaves D, Trigwell P. A scale for the assessment of hedonic tone the Snaith-Hamilton Pleasure Scale. *Br J Psychiatry.* 1995;167(1):99-103.
122. Gooding DC, Pflum MJ. The assessment of interpersonal pleasure: introduction of the Anticipatory and Consummatory Interpersonal Pleasure Scale (ACIPS) and preliminary findings. *Psychiatry Res.* 2014;215(1):237-243.
123. Treadway MT, Buckholtz JW, Schwartzman AN, Lambert WE, Zald DH. Worth the 'EEfRT'? The effort expenditure for rewards task as an objective measure of motivation and anhedonia. *PLoS One.* 2009;4(8):e6598.
124. Johnson MW, Bickel WK. Within-subject comparison of real and hypothetical money rewards in delay discounting. *J Exp Anal Behav.* 2002;77(2):129-146.
125. Carver CS, White TL. Behavioral inhibition, behavioral activation, and affective responses to impending reward and punishment: the BIS/BAS Scales. *Journal of Personality and Social Psychology.* 1994;67(2):319-333.
126. Watson D, O'Hara MW, Naragon-Gainey K, Koffel E, Chmielewski M, Kotov R, Stasik SM, Ruggero CJ. Development and validation of new anxiety and bipolar symptom scales for an expanded version of the IDAS (the IDAS-II). *Assessment.* 2012;19(4):399-420.
127. Forbush K, Wildes J, Hunt T. Gender norms, pschometric properties, and validity for the Eating PAthology Symptoms Inventory. *International Journal of Eating Disorders.* 2014;47:85-91.
128. Forbush K, Wildes J, Pollack L, Dunbar L, Luo D, Patterson J, Petruzzi K, Pollpeter L. Development and Validation of the Eating Pathology Symptoms Inventory (EPSI). *Psychological Assessment.* 2013;25(3):859-878.

129. Forbush K. Eating Pathology Symptoms Inventory (EPSI). In: Wade T, ed. *Encyclopedia of Feeding and Eating Disorders*. Singapore: Springer; 2017.
130. Marin RS, Biedrzycki RC, Firinciogullari S. Reliability and validity of the Apathy Evaluation Scale. *Psychiatry Res.* 1991;38(2):143-162.
131. Sullivan B, Payne TW. Affective disorders and cognitive failures: a comparison of seasonal and nonseasonal depression. *Am J Psychiatry.* 2007;164(11):1663-1667.
132. Broadbent DE, Cooper PF, FitzGerald P, Parkes KR. The Cognitive Failures Questionnaire (CFQ) and its correlates. *Br J Clin Psychol.* 1982;21 (Pt 1):1-16.
133. Kilbourn MR, Koeppe RA. Classics in Neuroimaging: Radioligands for the Vesicular Monoamine Transporter 2. *ACS Chem Neurosci.* 2019;10(1):25-29.
134. Boileau I, McCluskey T, Tong J, Furukawa Y, Houle S, Kish SJ. Rapid Recovery of Vesicular Dopamine Levels in Methamphetamine Users in Early Abstinence. *Neuropsychopharmacology.* 2016;41(4):1179-1187.
135. Chan GL, Holden JE, Stoessl AJ, Samii A, Doudet DJ, Dobko T, Morrison KS, Adam M, Schulzer M, Calne DB, Ruth TJ. Reproducibility studies with 11C-DTBZ, a monoamine vesicular transporter inhibitor in healthy human subjects. *J Nucl Med.* 1999;40(2):283-289.
136. Collantes M, Penuelas I, Alvarez-Erviti L, Blesa J, Marti-Climent JM, Quincoces G, Delgado M, Ecay M, Martinez A, Arbizu J, Rodriguez-Oroz MC, Obeso J, Richter JA. [Use of 11C-(+)-alpha -dihydrotetrabenazine for the assessment of dopaminergic innervation in animal models of Parkinson's disease]. *Rev Esp Med Nucl.* 2008;27(2):103-111.
137. Nag S, Jahan M, Toth M, Nakao R, Varrone A, Halldin C. PET Imaging of VMAT2 with the Novel Radioligand [(18)F]FE-DTBZ-d4 in Nonhuman Primates: Comparison with [(11)C]DTBZ and [(18)F]FE-DTBZ. *ACS Chem Neurosci.* 2021;12(24):4580-4586.
138. Okamura N, Villemagne VL, Drago J, Pejoska S, Dhamija RK, Mulligan RS, Ellis JR, Ackermann U, O'Keefe G, Jones G, Kung HF, Pontecorvo MJ, Skovronsky D, Rowe CC. In vivo measurement of vesicular monoamine transporter type 2 density in Parkinson disease with (18)F-AV-133. *J Nucl Med.* 2010;51(2):223-228.
139. Rusjan P, Mamo D, Ginovart N, Hussey D, Vitcu I, Yasuno F, Tetsuya S, Houle S, Kapur S. An automated method for the extraction of regional data from PET images. *Psychiatry Res.* 2006;147(1):79-89.
140. Duvernoy H. *The Human Brain: Surface, Blood Supply and Three Dimensional Sectional Anatomy*. New York: SpringerWien; 1999.
141. Talairach J, Tournoux P. *Co-Planar Stereotaxic Atlas of the Human Brain*. New York: Thieme Medical; 1988.
142. Mawlawi O, Martinez D, Slifstein M, Broft A, Chatterjee R, Hwang DR, Huang Y, Simpson N, Ngo K, Van Heertum R, Laruelle M. Imaging human mesolimbic dopamine transmission with positron emission tomography: I. Accuracy and precision of D(2) receptor parameter measurements in ventral striatum. *J Cereb Blood Flow Metab.* 2001;21(9):1034-1057.
143. Chiuccariello L, Houle S, Miler L, Cooke RG, Rusjan PM, Rajkowska G, Levitan RD, Kish SJ, Kolla NJ, Ou X, Wilson AA, Meyer JH. Elevated monoamine oxidase a binding during major depressive episodes is associated with greater severity and reversed neurovegetative symptoms. *Neuropsychopharmacology.* 2014;39(4):973-980.

144. Studholme C, Hill D, Hawkes D. An Overlap Invariant Entropy Measure of 3D Medical Image Alignment. *Pattern Recognition.* 1999;32:71-86.
145. Muller-Gartner HW, Links JM, Prince JL, Bryan RN, McVeigh E, Leal JP, Davatzikos C, Frost JJ. Measurement of radiotracer concentration in brain gray matter using positron emission tomography: MRI-based correction for partial volume effects. *J Cereb Blood Flow Metab.* 1992;12(4):571-583.
146. Boileau I, Houle S, Rusjan PM, Furukawa Y, Wilkins D, Tong J, Selby P, Wilson AA, Kish SJ. Influence of a low dose of amphetamine on vesicular monoamine transporter binding: a PET (+)[11C]DTBZ study in humans. *Synapse.* 2010;64(6):417-420.
