# Supplementary material for: Loss of vesicular monoamine transporter 2 in striatum of long COVID and relationship to neuropsychiatric symptoms
Source: eBioMedicine. 2026 Jul 10;130:106339. doi: 10.1016/j.ebiom.2026.106339 (PMC13476897; doi:10.1016/j.ebiom.2026.106339)
Supplement: Supplementary file 2 [file mmc2.docx]

**SUPPLEMENTAL APPENDIX**

Loss of Vesicular Monoamine Transporter 2 in Striatum of Long COVID and Relationship to Neuropsychiatric Symptoms

**Supplementary Figures**

**Figure S1.** Mean time-activity curves of (+)[^11^C]DTBZ radioactivity in the reference region over 60 minutes post-injection.

**Figure S2.** Pearson correlation coefficients (r) between regional (+)[^11^C]DTBZ BP_ND_ and depression severity in long COVID (n = 24).

**Figure S3.** Pearson correlation coefficients (*r*) between regional (+)[^11^C]DTBZ BP_ND_ and date of acute COVID-19 prior to onset of long COVID and duration since acute COVID-19 in long COVID (n = 24).

**Figure S4.** Pearson correlation coefficients (r) between regional (+)[^11^C]DTBZ BP_ND_ and plasma markers in long COVID (n = 24).

**Supplementary Tables**

**Table S1.** (+)[^11^C]DTBZ PET Scan Parameters of Main Participant Groups

**Table S2.** A Comparison of Symptom Measures Between Those with Long COVID and Those Recovered Well from COVID-19

**Table S3.** Relationship of Regional (+)[^11^C]DTBZ BP_ND_ with Neurocognitive Test Scores in Long COVID

**Table S4.** Unilateral (+)[^11^C]DTBZ BP_ND_ in 24 Long COVID Cases Compared to 24 Age-Matched Healthy Controls

**Supplementary Methods**

**Supplementary Results**

**Figure S1. Mean time-activity curves of (+)[^11^C]DTBZ radioactivity in the reference region over 60 minutes post-injection.**

Error bars indicate standard deviation. Blue error bars are present but beneath other error bars.

**Figure S2. Pearson correlation coefficients (*r*) between regional (+)[^11^C]DTBZ BP_ND_ and depression severity in long COVID (n = 24).**

**Figure S3. Pearson correlation coefficients (*r*) between regional (+)[^11^C]DTBZ BP_ND_ and date of acute COVID-19 prior to onset of long COVID and duration since acute COVID-19 in long COVID (n = 24).**

Dashed lines in Figure S4A represent onset of significant predominant variants in Ontario.

**Figure S4. Pearson correlation coefficients (*r*) between regional (+)[^11^C]DTBZ BP_ND_ and plasma markers in long COVID (n = 24).**

NfL = neurofilament light chain. HVA = homovanillic acid. DOPAC = 3,4-Dihydroxyphenylacetic acid.

**Table S1. (+)[^11^C]DTBZ PET Scan Parameters of Main Participant Groups**

|  | **Long COVID (n=24)** | **Age-Matched Healthy (n=24)** | ***P* value** |
| --- | --- | --- | --- |
| **(+)[^11^C]DTBZ Specific Activity (mCi/μmol), Mean (SD)** | 2040.5 (910.2) | 2365.0 (1153.3) | .30 **^a^** |
| **Cerebellar Cortex TAC AUC (MBq·min/cc), Mean (SD)** | 859.7 (161.5) | 879.6 (210.3) | .71 ^b^ |

There was no significant difference in (+)[^11^C]DTBZ specific activity injected or the area under curve of the reference tissue (cerebellar cortex) time-activity curve.

TAC = Time-Activity Curve. AUC = Area Under Curve. SD = Standard Deviation.

^a^ Welch’s t-test

^b^ Cerebellum standardized uptake value was also not significantly different between groups

**Table S2. A Comparison of Symptom Measures Between Those with Long COVID and Those Recovered Well from COVID-19**

| **Symptom Measures** | **Long COVID (n=24)** | **Healthy Recovered Well from COVID-19 (n=24)** |  |
| --- | --- | --- | --- |
|  | **Mean (SD)** | **Mean (SD)** | ***P* value ^c^** |
| **Marin Apathy Evaluation Scale ^a^** | 45.6 (5.7) | 23.9 (4.0) | 2×10^-16^ |
| **HVLT-R Delayed Recall ^b^** | 47.6 (10.8) | 58.5 (3.4) | 6×10^-5^ |
| **Cognitive Failure Questionnaire ^a^** | 39.4 (15.2) | 22.1 (10.9) | 5×10^-5^ |
| **FTT (DH) ^b^** | 34.0 (8.5) | 44.7 (9.3) | 1×10^-4^ |
| **FTT (NDH) ^b^** | 32.8 (7.9) | 44.0 (9.7) | 1×10^-4^ |
| **Stroop Word ^b^** | 46.0 (8.2) | 46.1 (10.9) | 0.98 |

Across neuropsychiatric assessments and clinical scales, 24 long COVID individuals showed more severe symptoms in FTT (DH and NDH), HVLT-R Delayed Recall, Cognitive Failure Questionnaire, and Marin Apathy Evaluation Scale than the 24 healthy controls recovered well after COVID-19.

FTT = Finger Tapping Test. DH = dominant hand. NDH = non-dominant hand. HVLT-R = Hopkins Verbal Learning Test-Revised.

^a^ Raw scores used for analysis

^b^ T-scores used for analysis

^c^ Welch’s t-test

**Table S3. Relationship of Regional (+)[^11^C]DTBZ Binding Potential with Neurocognitive Test Scores in Long COVID**

|  |  | | **Regions of Interest** | |  | |
| --- | --- | --- | --- | --- | --- | --- |
|  | **Ventral Striatum** | | **Dorsal Putamen** | | **Dorsal Caudate** | |
|  | ***r or ρ* (95% CI)** | ***P*** | ***r or ρ* (95% CI)** | ***P*** | ***r or ρ* (95% CI)** | ***P*** |
| **Anhedonia** |  |  |  |  |  |  |
| **SHAPS** | -0.005 (-0.45 to 0.34)^b^ | .99 | 0.17 (-0.23 to 0.54)^b^ | .41 | 0.09 (-0.29 to 0.46)^b^ | .66 |
| **Apathy** |  |  |  |  |  |  |
| **AES** | -0.54 (-0.86 to -0.13)^b^ | **.0069** | -0.094 (-0.48 to 0.32) | 0.66 | -0.14 (-0.52 to -0.28) | .51 |
| **Psychomotor Speed** |  |  |  |  |  |  |
| **FTT (DH)^a^** | 0.16 (-0.26 to 0.53) | .46 | 0.51 (0.14 to 0.76) | **.010** | 0.35 (-0.07 to 0.66) | .10 |
| **FTT (NDH) ^a^** | 0.35 (-0.06 to 0.66) | .095 | 0.47 (0.08 to 0.74) | **.020** | 0.41 (0.008 to 0.70) | .**046** |
| **Executive Function** |  |  |  |  |  |  |
| **CFQ** | -0.52 (-0.76 to -0.15) | **.009** | -0.36 (-0.67 to 0.05) | .084 | -0.16 (-0.53 to 0.26) | .45 |
| **CPT Omission Error** | -0.19 (-0.59 to 0.23)^b^ | .37 | 0.02 (-0.40 to 0.43)^b^ | .94 | -0.06 (-0.49 to 0.35)^b^ | .78 |
| **CPT Commission Error** | -0.10 (-0.51 to 0.31)^b^ | .65 | 0.17 (-0.25 to 0.52)^b^ | .43 | 0.06 (-0.33 to 0.43)^b^ | .78 |
| **WCST Preservative Errors ^a^** | -0.03 (-0.43 to 0.38) | .90 | 0.33 (-0.15 to 0.72)^b^ | .12 | -0.19 (-0.55 to 0.23) | .37 |
| **WCST Conceptual Level ^a^** | -0.00 (-0.42 to 0.44)^b^ | .99 | 0.13 (-0.25 to 0.48)^b^ | .54 | -0.03 (-0.43 to 0.35)^b^ | .88 |
| **HVLT-R Total Recall ^a^** | 0.48 (0.09 to 0.74) | **.018** | 0.35 (-0.06 to 0.66) | .093 | 0.47 (0.08 to 0.73) | **.021** |
| **HVLT-R Delayed Recall ^a^** | 0.49 (0.11 to 0.75) | **.014** | 0.53 (0.16 to 0.77) | **.0081** | 0.58 (0.23 to 0.80) | **.0029** |
| **HVLT-R Retention ^a^** | 0.19 (-0.23 to 0.55) | .37 | 0.34 (-0.08 to 0.65) | .11 | 0.33 (-0.09 to 0.65) | .12 |
| **BVMT-R Total Recall ^a^** | 0.15 (-0.28 to 0.54)^b^ | .49 | 0.001 (-0.47 to 0.44)^b^ | .99 | 0.38 (-0.03 to 0.69)^b^ | .071 |
| **BVMT-R Learning ^a^** | -0.24 (-0.59 to 0.18) | .26 | -0.25 (-0.59 to 0.17) | .24 | -0.52 (-0.84 to -0.11)^b^ | **.0096** |
| **BVMT-R Delayed Recall ^a^** | 0.03 (-0.37 to 0.42)^b^ | .88 | -0.07 (-0.45 to 0.28)^b^ | .73 | 0.06 (-0.31 to 0.43)^b^ | .80 |

Data are correlations between regional (+)[^11^C]DTBZ BP_ND_ values and neuropsychological test scores in long COVID (n = 24).

SHAPS = Snaith-Hamilton Pleasure Scale. FTT = Finger Tapping Test. DH = dominant hand. NDH = non-dominant hand. AES = Marin Apathy Evaluation Scale. CFQ = Cognitive Failure Questionnaire. CPT = Continuous Performance Test. WCST = Wisconsin Card Sorting Test. HVLT-R = Hopkins Verbal Learning Test-Revised. BVMT-R = Brief Visuospatial Memory Test-Revised.

^a^ T-scores used for analysis

^b^ Spearman rank correlation coefficient (*ρ*) and relevant 95% CI and *P* values reported due to non-normal distribution of residuals (Shapiro-Wilk test, p < .05). All other correlations use Pearson's *r*.

**Table S4. Unilateral (+)[^11^C]DTBZ BP_ND_ in 24 Long COVID Cases Compared to 24 Age-Matched Healthy Controls**

|  | **(+)[^11^C]DTBZ BP_ND_, mean (SD)** | |  |  |
| --- | --- | --- | --- | --- |
| **Regions of Interest** | **Long COVID (n=24)** | **Age-Matched Healthy (n=24)** | ***P* value** ^a^ **(Group)** | ***P* value** ^a^ **(Laterality)** |
| **Ventral Striatum (Left)** | 1.21 (0.35) | 1.46 (0.24) | .0027 | .76 |
| **Ventral Striatum (Right)** | 1.20 (0.30) | 1.43 (0.28) | .0061 |  |
| **Dorsal Putamen (Left)** | 1.92 (0.30) | 2.25 (0.18) | 1×10^-4^ | .97 |
| **Dorsal Putamen (Right)** | 1.88 (0.38) | 2.21 (0.20) | 1×10^-4^ |  |
| **Dorsal Caudate (Left)** | 1.72 (0.28) | 2.06 (0.25) | 6×10^-4^ | .79 |
| **Dorsal Caudate (Right)** | 1.71 (0.37) | 2.04 (0.24) | 1×10^-4^ |  |

Across unilateral regions measured, (+)[^11^C]DTBZ BP_ND_ is significantly lower in 24 long COVID cases compared to 24 age matched healthy controls (linear mixed effects model, effect size 1.05, *P* = 2×10^-5^). No effect of laterality was found for any region.

^a^ Linear mixed effects model with group and laterality as fixed factors; *P* values reflect comparisons of estimated marginal means

**Supplementary Methods**

In addition, as an exploratory analysis, (+)[^11^C]DTBZ BP_ND_ was measured in unilateral striatal regions and laterality was assessed through application of a linear mixed effects model.

Additional enrolment criteria for both groups included age 18 to 75; good general physical health with no active medical conditions based on self-report (except migraine or post acute sequelae of COVID symptoms (PASC) in the long COVID group); use of medications or herbal products or natural health products with central nervous system effects in past 2 weeks based on self-report; presence of cigarette smoking in the past two months, based on self-report and urine cotinine screening; lifetime diagnosis of severe Substance or Alcohol Use Disorder, or diagnosis of mild to moderate Substance or Alcohol Use Disorder in the past two years, based on self-report and verified by the Research Version of Structured Clinical Interview for Diagnostic and Statistical Manual-5 (SCID-5-RV); use of recreational drugs, including marijuana, in the past two months, based on self-report and verified by the Research Version of Structured Clinical Interview for Diagnostic and Statistical Manual-5 (SCID-5-RV); positive urine drug or cotinine screen at any timepoint during the study; history of psychotic symptoms secondary to schizophrenia, schizophreniform, bipolar disorder, or brief psychotic disorder prior to COVID-19, based on self-report and verified by the Research Version of Structured Clinical Interview for Diagnostic and Statistical Manual-5 (SCID-5-RV). History of psychotropic medication use was exclusionary for healthy participants and use of stimulant medication, antipsychotics or antidepressants in the previous month (6 weeks for fluoxetine) was exclusionary for long COVID cases.

**Supplementary Results**

In an exploratory analysis, a linear mixed effects model including each unilateral region as a repeated variable found that the effect of group was similar (Supplementary Table S4). Also, within each region, a linear mixed model was also applied, evaluating laterality and group as factors. Group was persistently significant, but no effect of laterality was found for any region (Supplementary Table S4).
